# DNA barcoding the ichthyofauna of the Beibu Gulf: Implications for fisheries management in a seafood market hub

**DOI:** 10.1002/ece3.10822

**Published:** 2023-12-11

**Authors:** Changping Jiang, Murong Yi, Zhisen Luo, Xiongbo He, Hung‐Du Lin, Nicolas Hubert, Yunrong Yan

**Affiliations:** ^1^ College of Fisheries Guangdong Ocean University Zhanjiang China; ^2^ Southern Marine Science and Engineering Guangdong Laboratory (Zhanjiang) Zhanjiang China; ^3^ Guangdong Provincial Engineering and Technology Research Center of Far Sea Fisheries Management and Fishing of South China Sea Guangdong Ocean University Zhanjiang China; ^4^ The Affiliated School of National Tainan First Senior High School Tainan Taiwan; ^5^ Institut de Recherche pour le Développement, UMR 226 ISEM (IRD, UM, CNRS) Université de Montpellier Montpellier France

**Keywords:** conservation, DNA‐based species delimitation, fisheries management, stock collapse, Western Pacific Ocean

## Abstract

The Beibu Gulf in China is situated in the tropics, in the western Pacific Ocean. It is an emblematic region combining proximity to a marine biodiversity hotspot and a major seafood hub. Intensification of marine fishing and ocean warming led to a drastic decline in fish populations in the Beibu Gulf during the last decades. This situation urges the development of molecular resources of the Beibu Gulf fish fauna in order to enable automated molecular identifications at the species level for next‐generation monitoring. With this objective, we present the results of a large‐scale campaign to DNA barcode fishes of the Beibu Gulf. We successfully generated 789 new DNA barcodes corresponding to 263 species which, together with 291 sequences mined from Genbank and BOLD, resulted in a reference library of 1080 sequences from 285 species. Based on the use of four DNA‐based species delimitation methods (BIN, ASAP, mPTP, mGMYC), a total of 285 Molecular Operational Taxonomical Units (MOTUs). A single case of cryptic diversity was detected in *Scomberomorus guttatus* and a single species pair was not captured by delimitation methods. Intraspecific K2P genetic distances averaged 0.36% among sequences within species, whereas K2P genetic distances among species within genera averaged 6.96%. The most speciose families in open water trawling differ from those at fish market, and discrepancies with historical data are discussed in the light of recently documented stock collapses.

## Graphical abstract

The fish fauna of the beibu gulf, one of the largest sea food hubs in Southeast Asia, is characterized here by DNA barcoding. The 1080 DNA barcodes analyzed show concordance between morphological identifications and DNA‐based species delimitation. Lower level of diversity detected here compared to historical observations is concordant with the stock collapses previously described in the literature for the area. This study opens new perspectives by enabling next‐generation monitoring of the 285 species DNA barcoded here.

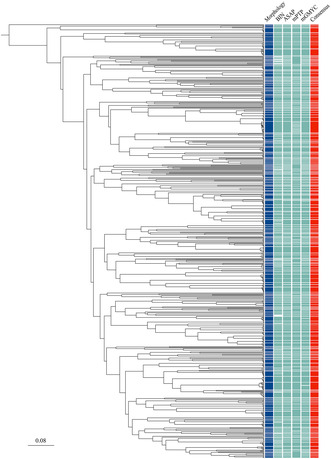

## 1 INTRODUCTION

To date, 36,552 fish species have been described during more than two centuries of inventory of species, out of which 50% live in the marine realm (Fricke et al., 2023; Miller, 2021; Rabosky et al., 2018). In the past few decades, the rise of global temperature, overexploitation of natural resources, and development of human activities led to increasing levels of threats to biological resources (Hoffmann et al., 2010; Myers et al., 2000; Schipper et al., 2008). Marine biological resources are no exception and the vast array of anthropogenic pressures threatening it has attracted the attention of numerous specialists (Cinner et al., 2016; Thiault et al., 2019). However, the ability to anticipate and mitigate biodiversity loss is not only of prime importance for conservation purposes but also has major implications for food security, livelihood, and global economy (Lal, 2004; Lobell et al., 2008). Under the concomitant influence of anthropogenic pressures and global changes, the global decline of marine fish species is gradually accelerating, compromising both the sustainability of marine resource harvesting and the resilience of marine ecosystems (Bryndum‐Buchholz et al., 2020; Mullon et al., 2005; Thiault et al., 2019; Worm et al., 2006).

Located at 17–22° N and 105–110° E in the western Pacific Ocean, the Beibu Gulf is a tropical hotspot of marine biodiversity and also a market hub of seafood in Southeast Asia. Originally known as the Gulf of Tonkin, the Beibu Gulf is China's four most renowned traditional fishing areas (Qiu et al., 2008). With an average depth of 38 meters and an area of 128,800 km^2^, it is situated northwest of the South China Sea on the continental shelf. The gulf exhibits a diversity of coastal habitats including mangroves, coral reefs, and estuaries which are supplied by numerous rivers from China and Vietnam, such as the Red River and the Nanliu River (Chen et al., 2009; Qiu et al., 2008). The gulf also exhibits a diversity of physical habitats with two gradients of surface water temperature, one from south to north and a second from the nearshore to the bay (Fu et al., 2012; Qiao & Lin, 2007).

The environmental heterogeneity of the Beibu Gulf likely accounts for the high diversity of marine fishes recorded in the area with 646 species (Sun et al., 2010). Nevertheless, the diversity of fishes in the Beibu Gulf might be underestimated as no compilation of fish diversity has been produced recently. With the development of molecular systematics, the taxonomic system of fish species has undergone significant changes, making it necessary to integrate morphological and molecular results for fish species in the Beibu Gulf. Trends in fisheries are traditionally surveyed using biomass‐based methods, which are informative at large spatial and temporal scales (Cinner et al., 2013, 2016; Maire et al., 2016). However, surveying fisheries at smaller spatial and taxonomic scales requires accurate taxonomic knowledge. Unfortunately, over‐exploitation, illegal fishing, environmental pollution, and biological invasion induced a quick drop in fishery resources and fishing over the past decade, a trend calling for new approaches to monitor fisheries in the Beibu Gulf at multiple spatial scales (Qiu et al., 2008; Sun & Lin, 2004; Wang et al., 2010; Zhang et al., 2020).

DNA barcoding, the use of a standardized gene fragment as an internal species tag, aims at enabling automated molecular species identifications (Hebert, Cywinska, et al., 2003; Hebert, Ratnasingham, & deWaard, 2003). This already proven approach for fishes opened new perspectives by improving taxonomic knowledge in tropical regions (Arida et al., 2021; Pereira et al., 2013; Shen et al., 2019; Sholihah et al., 2020; Sonet et al., 2019) and by allowing automated identifications (April et al., 2011; Hubert et al., 2008). As such, DNA barcoding offers numerous applications in the management of marine fisheries such as the detection of market substitution (Carvalho et al., 2015; Hanner et al., 2011; Wong & Hanner, 2008), and the recognition of untapped diversity among commercially important species (Delrieu‐Trottin et al., 2019, 2020; Durand et al., 2017; Jaafar et al., 2012). According to existing literature, DNA barcoding can be used for rapid and effective identification of fish species. However, since its first use in the South China Sea region in 2012, the Beibu Gulf has been almost untouched and awaits a campaign to DNA barcode its fish diversity (Hou et al., 2018, 2022; Zhang & Hanner, 2012).

Here, we present the results of a large‐scale DNA barcoding campaign of the Beibu Gulf ichthyofauna aiming at providing a validated DNA barcode reference library to enable automated fish species identification. This approach not only accounts for potential cryptic diversity but also helps to establish sustainable genetic resources for fisheries monitoring and food traceability. The Beibu Gulf hosts some of the most productive fisheries in the South China Sea, which constitute important socioeconomic drivers of the region (Qiu et al., 2008). As such, the Beibu Gulf exemplifies contemporary trends in major seafood market hub in Southeast Asia, and the DNA barcoding campaign developed here is expected to provide valuable genetic resources for fisheries management elsewhere.

## 2 MATERIALS AND METHODS

### 2.1 Sampling and morphological identification

**FIGURE 1:**
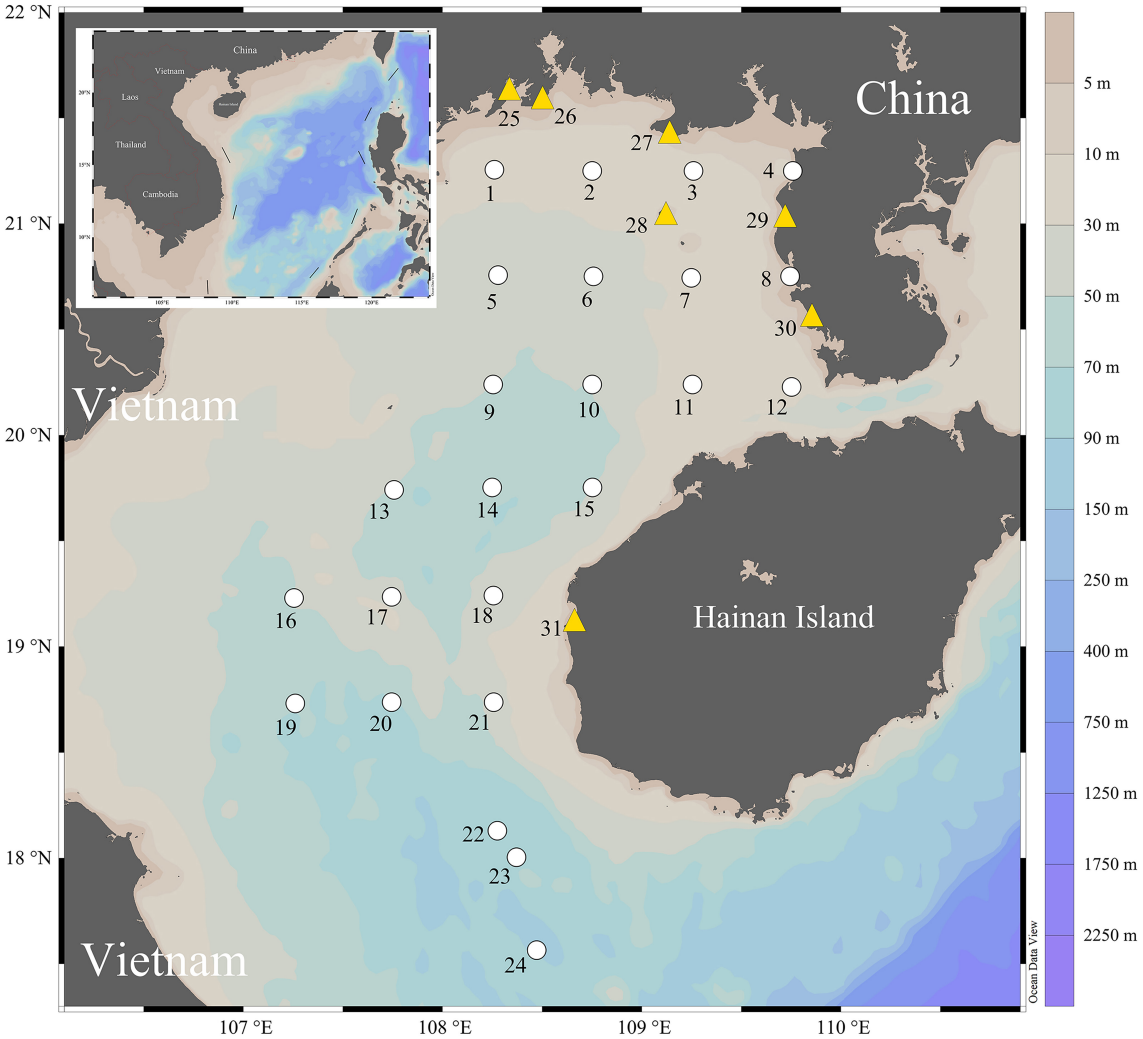
Collection sites for the newly generated DNA barcode records of the present study. Symbols represent different types of sites; white circle correspond to the 24 stations in open waters; yellow triangles to the 7 fish markets visited (Fangchenggang, Qisha, Beihai, Weizhou Island, Jianghong, Wushi, and Basuo). This map was created with Ocean Data View.

Specimens were collected at 24 locations offshore using bottom trawler nets (maximum mesh size of 50 mm) and complemented by visiting fish stalls at 7 harbors in the Beibu Gulf from September 2, 2014, to September 1, 2022 (Figure 1). All samples involve common economic fish species as well as bycatch of low‐value species (commonly used as feedmeat for aquaculture). Offshore sites were defined using a grid covering the ecological and physical diversity of the environmental conditions in the gulf and trawling was conducted for half an hour at each site. For each specimen, identifiers, collectors, collecting date, region, geographic coordinates, and photograph were recorded (Table S1). Preliminary identifications to the species level were conducted in the field following the Systematic Synopsis of Chinese Fishes (Chen & Zhang, 1982), Fishes of Beibu Gulf (Liu et al., 2016), and the Atlas Guide to Economic Fishes of South China Sea (Yan et al., 2020), and upper‐rank systematic follow Fishes of the Word (Nelson et al., 2016). Specimens were individually labeled and photographed, and a muscle biopsy of approximately 50 mg was performed on the right side. All specimens were soaked in a 20% formalin solution for 3–7 days before being washed with water and transferred to 75% ethanol for long‐term preservation. Voucher specimens and tissue samples were deposited at the Guangdong Ocean University.

### 2.2 DNA extraction, amplification and sequencing

DNA was extracted using the FastPure® Cell/Tissue DNA Isolation Mini Kit methodology using the manufacturer's specification. A fragment of 652 bp of the mitochondrial COI gene was amplified using the universal primers FishF_1_/FishR_1_ and FishF_2_/FishR_2_ (Ward et al., 2005). Amplifications were conducted in the following conditions: 3.5 μL of ultrapure water, 2.5 μL of MgCl_2_ (5 mM), 3.5 μL of buffer, 1 μL of forward and reverse primers, 12.5 μL of Taq polymerase and 5 μL of genomic DNA. Amplifications employed the following thermocycling regime: initial denaturation at 92°C for 5 min followed by 33 cycles including denaturation at 92°C for 45 s, annealing at 50°C for 60 s and extension at 72°C for 60 s, followed by a final extension step at 72°C for 10 min. Sanger sequencing was conducted at Sangon Biotech company, and reads were assembled and edited using Sequencher 5.4.5. The primer pairs FishF1/FishR1 and FishF2/FishR2 resulted in sequences of 655 and 652 bp, respectively. Sequences analyzed in the present study were deposited in Genbank (accession numbers OQ552889–OQ553649, OQ559023–OQ559041, and OR523579–OR523587).

### 2.3 Genetic distance analyses

Sequences were aligned using MAFFT (Katoh & Standley, 2013), which was also used to translate nucleotidic into amino‐acid sequences to check the occurrence of potential stop codons. A matrix of pairwise Kimura‐2‐Parameters genetic distance (Kimura, 1980) was calculated using the R package Ape 5.6‐2 (Paradis & Schliep, 2019). Distances to the nearest phylogenetic neighbor and the maximum intra‐specific distances were then extracted from the pairwise genetic distance matrix using the R package Spider 1.5.0 (Brown et al., 2012). We further checked for a DNA barcoding gap in our data (Meyer & Paulay, 2005). Instead of considering potential overlaps in the distribution of each distance independently, we examined the relationships between both distances on an individual basis (Blagoev et al., 2015). Plots were created using the R package ggplot2 3.3.6 (Wickham, 2009). Finally, a Neighbor joining (NJ) tree was constructed using K2P genetic distances with the R package ggtree2 3.2.1 (Yu, 2020), for a visual inspection of genetic distances and DNA barcode clusters (Figure S1).

### 2.4 Species delimitation

Species identification was verified by blasting newly generated sequences to GenBank. If a discordance between identification and blast results were detected, specimens were re‐examined and secondary morphological identifications were conducted. The final identification to the species level was established according to morphological characters. DNA barcoding delimitation approaches were implemented here in order to produce a robust delimitation scheme based on a majority rule consensus. This approach is increasingly used to overcome differences among algorithms regarding unequal sampling among lineages (Chen et al., 2022; Delrieu‐Trottin et al., 2020; Kekkonen & Hebert, 2014; Limmon et al., 2020; Shen et al., 2019; Sholihah et al., 2020). A total of four different methods were used: (1) Refined Single Linkage, implemented in Barcode of Life Database (BOLD) to produce Barcode Index Numbers (BINs) (Ratnasingham & Hebert, 2013), (2) Assemble Species by Automatic Partitioning (ASAP; Puillandre et al., 2021), (3) Multi‐rate (mPTP) Poisson Tree Process in the standalone program mPTP_0.2.3 (Kapli et al., 2017; Zhang et al., 2013), and (4) The General Mixed Yule‐Coalescent model in its multi‐rate version (mGMYC) using the R package Splits 1.0‐19 (Fujisawa & Barraclough, 2013). The final delimitation was established based on a majority rule consensus. For the sake of clarity, lineages identified using morphological characters are refer to as species, and lineages delimited by the species delimitation analyses are referred to as Molecular Operational Taxonomic Units (MOTUs).

Both Refined Single Linkage (RESL) and ASAP use DNA alignments as input, and sequences were submitted to the webserver of BOLD and ASAP for delimitation analysis respectively. For PTP analyses, a Maximum Likelihood (ML) tree was generated using the R package Phangorn 2.8.1 (Schliep, 2011) with a GTR + R8 + F substitution model. Finally, the ultrametric and fully resolved tree required by GMYC analyses was reconstructed using the Bayesian approach implemented in BEAST 2.4.8 (Bouckaert et al., 2014). Two Markov chains of 50 million each were run independently using a Yule pure birth model tree prior, a strict‐clock of 1.2% of genetic distance per million years (Bermingham et al., 1997), and a GTR + I + Γ substitution model. Trees were sampled every 10,000 states after an initial burn‐in period of 10 million. Both runs were first checked for statistical robustness (ESS > 200) using Tracer 1.7.1 (Rambaut et al., 2018) and further combined using LogCombiner 2.4.8. The maximum credibility tree was established using TreeAnnotator 2.4.7 (Bouckaert et al., 2014). Sequences were collapsed into haplotypes prior to Bayesian analyses using the ALTER webserver (http://www.sing‐group.org/ALTER/).

Finally, we checked the sampling coverage of the present survey at the species and MOTUs level by computing the sequence accumulation curve with by R package vegan (Oksanen et al., 2012).

## 3 RESULTS

**FIGURE 2:**
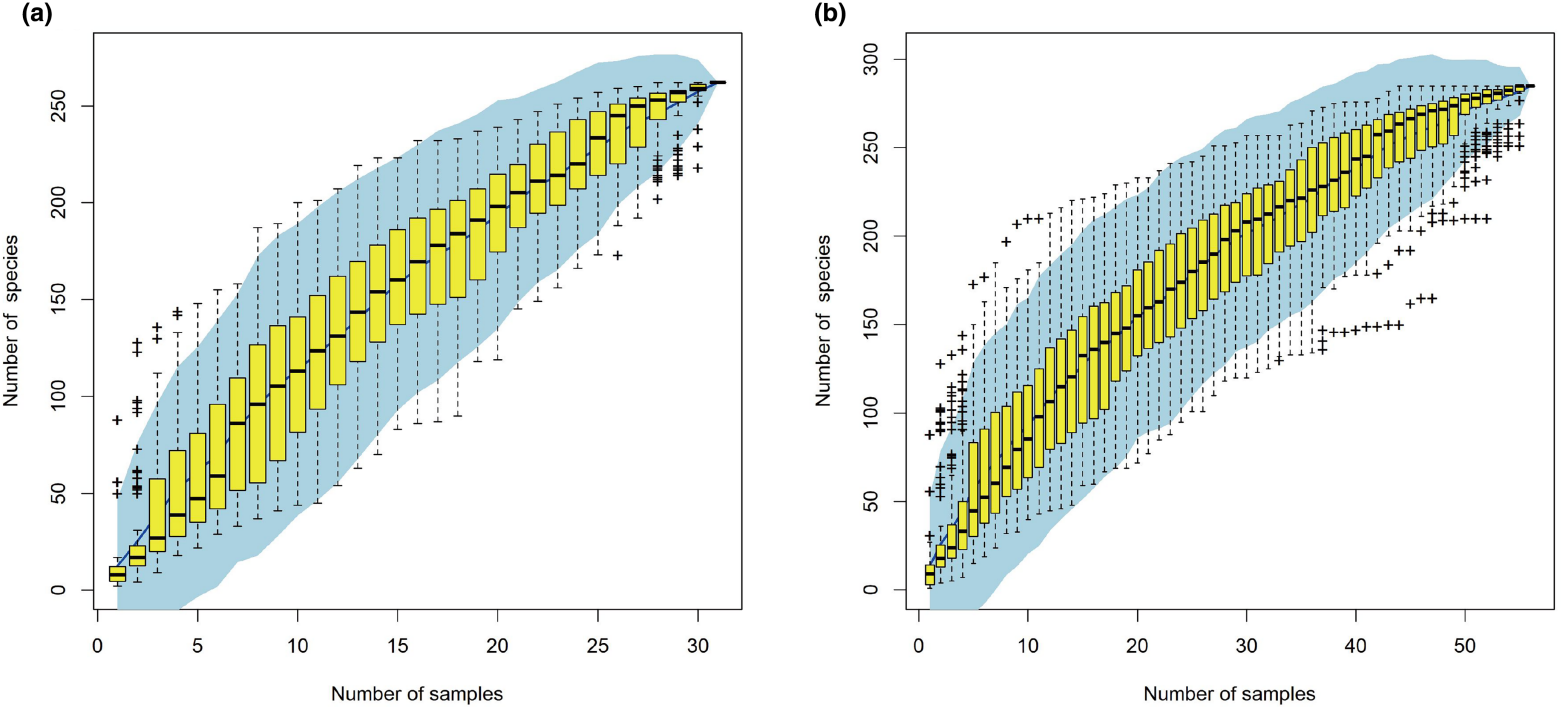
Species accumulation curves. (a) dataset including the 789 newly generated DNA barcode records; (b) dataset including the 1080 DNA barcode records including newly generated sequences and sequences mined in. BOLD. The light blue area corresponds to the 95% confidence interval. Yellow boxes are the interquartile ranges, the central bold mark on each box indicates the median, and the bottom and top edges of the box indicate the 25th and 75th percentiles, respectively. Outliers are plotted individually using the “+” symbol.

The trawling sessions yielded 375 specimens belonging to 122 species from 59 families and 18 orders, and visits to the fish stalls resulted in the collection of 414 specimens from 174 species belonging to 68 families and 15 orders (Table S1). Only 35 species corresponding to 24 families and 9 orders were observed both in trawling and fish stall samples (Table S1). A total of 789 new DNA barcode (COI sequences) sequences were successfully generated for 263 species out of the 833 samples analyzed. Amplification failures were randomly distributed among species, and all species were successfully sequenced. These new DNA barcode data represent 21 orders, 85 families, and 172 genera. Together with the 291 COI sequences mined in BOLD and originating from the Beibu Gulf, a DNA barcode reference library consisting of 1080 sequences from 285 species was assembled, which represent 21 orders, 88 families, and 185 genera (Table S1, Figure S1). The number of sequences per species varied from 1 to 23, with an average of 4 sequences per species. Compared to historical records, only 42% of the species previously reported from the Beibu Gulf by Sun et al. (2010) were observed and collected during the present study (Table 1), and several orders were not observed including Pristiformes, Rajiformes, Lamniformes, Torpediniformes, Squatiniformes, Osmeriformes, Atheriniformes and Syngathiformes. The accumulation curve established for a newly generated set of DNA barcode records is not reaching a plateau (Figure 2a). However, the accumulation curve calculated for the entire dataset reached a plateau (Figure 2b), suggesting that a comprehensive sampling effort was achieved.

**TABLE 1:** Taxonomic coverage across orders, families, genera, and species of the present study for the orders reported to occur in the Beibu Gulf by Sun et al. (2010).

Order	Family	Genus	Species	Percentage	No.
Carcharhiniformes	4/2	7/3	12/3	25	4
Orectolobiformes	2/1	2/1	2/1	50	1
Pristiformes	1/0	1/0	1/0	–	0
Rajiformes	6/0	14/0	23/0	–	0
Lamniformes	1/0	1/0	1/0	–	0
Torpediniformes	1/0	1/0	2/0	–	0
Squatiniformes	1/0	1/0	1/0	–	0
Mugiliformes	1/1	3/4	3/5	100^a^	9
Osmeriformes	1/0	1/0	1/0	–	0
Anguilliformes	8/4	15/6	29/7	24	8
Atheriniformes	1/0	2/0	2/0	–	0
Aulopiformes	1/1	4/3	8/4	50	14
Beloniformes	3/2	9/2	16/3	19	11
Beryciformes	2/1	3/1	3/1	33	1
Clupeiformes	4/4	18/12	32/19	59	76
Elopiformes	1/1	1/1	1/1	100	1
Gadiformes	2/1	2/1	2/1	50	2
Gasterosteiformes	1/2	2/2	2/2	100	2
Lophiiformes	3/1	3/1	5/1	20.0	2
Ophidiiformes	1/1	3/3	4/3	75	3
Perciformes	60/46	181/95	331/168	51	539
Pleuronectiformes	8/5	20/11	42/14	33	27
Scorpaeniformes	13/5	39/14	52/18	35	55
Siluriformes	4/2	5/3	5/3	60	7
Syngnathiformes	3/0	6/0	10/0	–	0
Tetraodontiformes	6/2	25/6	35/6	17	24
Zeiformes	1/1	1/1	1/1	100	1
Albuliformes	0/1	0/1	0/1	^a^	1
Scombriformes	0/1	0/1	0/1	^a^	1
Total	140/85	370/172	626/263	42	789

*Note*: n1/n2, n1 is the total number of taxa occurring in the Beibu Gulf and n2 is the number of taxa included in the present study. Percentage is the portion of the Beibu Gulf diversity represented in the present study. No is the number of barcodes extracted in this study.

^a^ Grouped with Perciformes in the present study.

According to the last assessment of the International Union for Conservation of Nature (IUCN: https://www.iucnredlist.org/), it appears that among the 285 species detected here, one species is considered as critically‐endangered (CR), *Sphyrna lewini* (Griffith & Smith, 1834), two species are indicated as endangered (EN), *Evynnis cardinalis* (Lacepède, 1802), and *Argyrosomus japonicus* (Temminck & Schlegel, 1843), three species are indicated as vulnerable (VU), *Albula glossodonta* (Forsskål, 1775), *Carcharhinus limbatus* (Valenciennes, 1839), *Nemipterus virgatus* (Houttuyn, 1782), seven are considered as near‐threatened (NT) *Scomberomorus commerson* (Lacepède, 1800), *Trachurus japonicus* (Temminck & Schlegel, 1844), *Scoliodon macrorhynchos* (Bleeker, 1852), *Choerodon schoenleinii* (Valenciennes, 1839), *Acanthopagrus chinshira* Kume & Yoshino, 2008, *Harpadon nehereus* (Hamilton, 1822), *Chiloscyllium plagiosum* (Anonymous [Bennett], 1830), and 171 are indicated as least‐concern (LC; Table S2). *Oreochromis mossambicus* (Peters, 1852) was also observed during the present survey and is indicated as threatened by the IUCN, however, *O. mossambicus* is considered as threatened only in its native range in Africa and is considered as invasive elsewhere.

**FIGURE 3:**
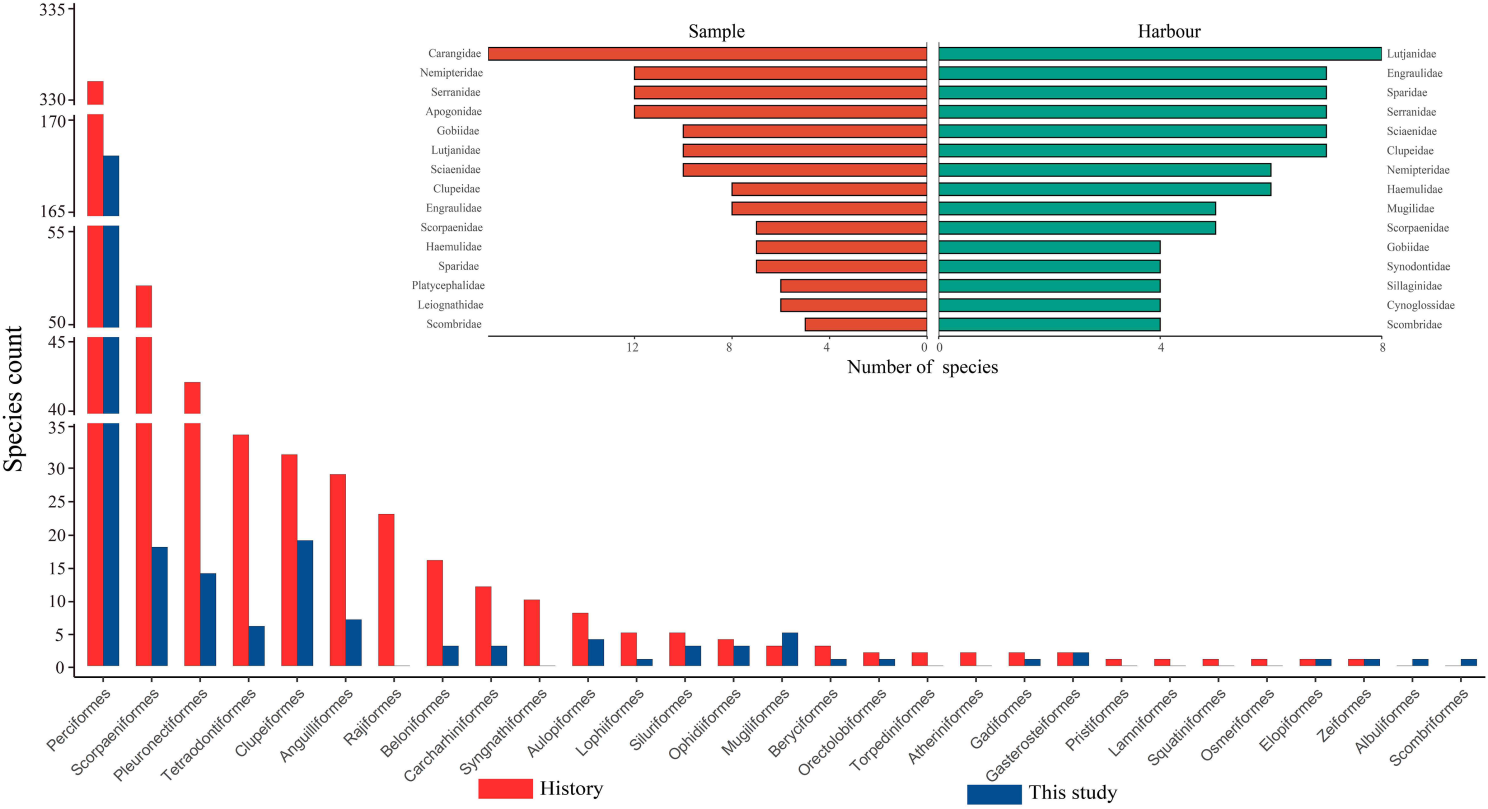
Species count ranked per family for historical records (Sun et al., 2010) in red and present study in blue. Upper panel, the top 15 families with the highest number of species in trawling (sample) and fish market (harbor).

According to Fishbase (Froese & Pauly, 2020), 83 species currently lack information. According to the Fish Database of Taiwan (https://fishdb.sinica.edu.tw/), 196 species are of commercial interest and 89 species are of low economic value. These 285 species display a great diversity of habitat preferences including for instance reef‐associated (e.g., Pomacanthidae, Fistulariidae, Holocentridae, and Ephippidae), demersal (e.g., Lophiidae, Cynoglossidae, and Paralichthyidae), and benthopelagic (e.g., Gempylidae, Nomeidae, and Stromateidae) families (Table S2). Of the families sampled during the trawling sessions, the family Carangidae display the highest diversity with 18 species (Figure 3). By contrast, the family Lutjanidae had the highest number of species at fish stalls in harbors with nine species. These two families were then followed by the families Clupeidae, Sciaenidae, Serranidae, Sparidae, and Engraulidae, with seven species, and many of which were present both in trawling collects and fish stalls.

**FIGURE 4:**
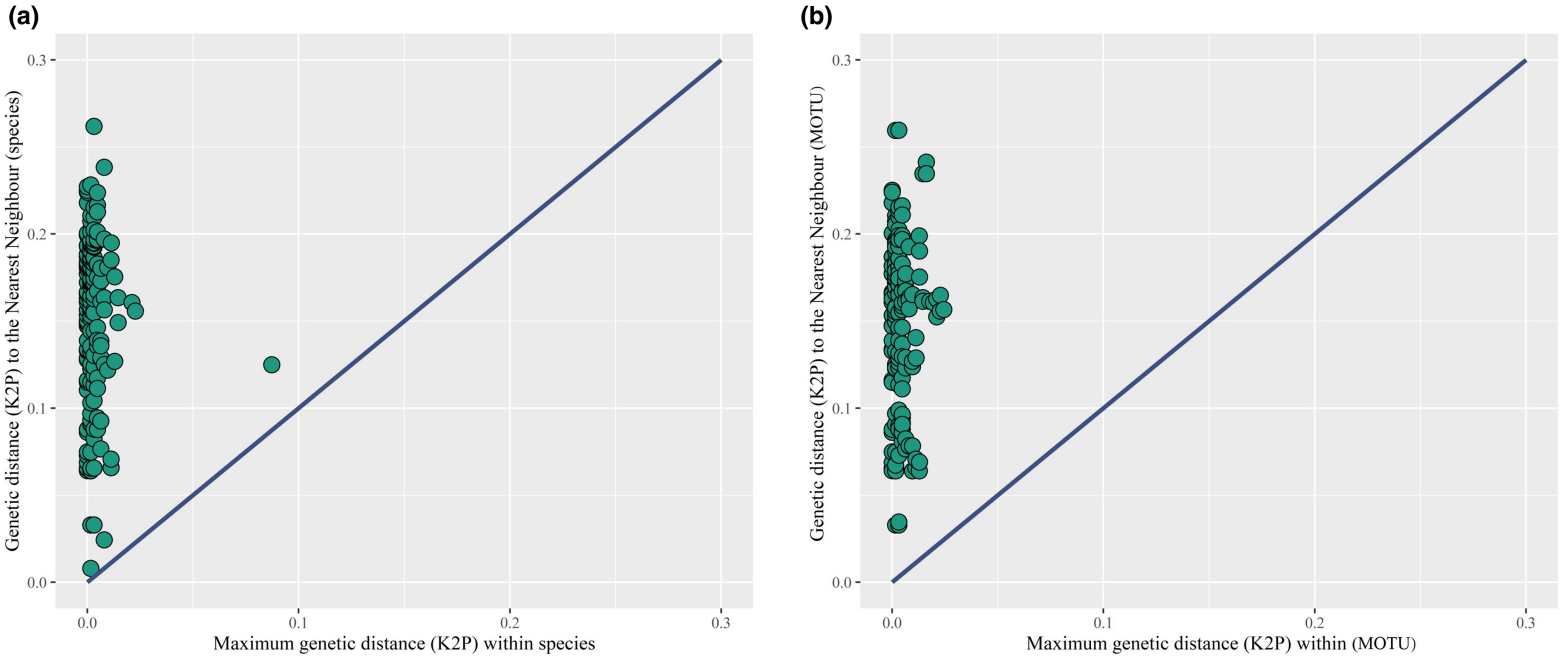
Relationships between maximum intraspecific and nearest neighbor K2P genetic distances. (a) Relationship between maximum intraspecific and nearest neighbor K2P genetic distances among species. (b) Relationship between maximum intraspecific and nearest neighbor K2P genetic distance among MOTUs.

K2P genetic distances between congeneric species averaged 0.0696, ranging from 0.00 to 0.283 (Champsodontidae). K2P distances within the family averaged 0.15, ranging from 0.00 to 0.3398 (Ophidiiformes) (Table 2). The maximum K2P intraspecific distance ranged between 0 and 0.0864 in *Scomberomorus guttatus* (Bloch & Schneider, 1801) with an average of 0.0036, whereas the nearest neighbor K2P distance ranged between 0.0077 and 0.2641 (Table S3), with an average of 0.1496. The ratio between nearest and maximum intraspecific K2P distances ranged between 0 and 134.97. No species with a nearest neighbor distance exceeding the maximum intraspecific distance was detected (Figure 4). Along the same line, no haplotype sharing or nearest neighbor genetic distance of 0 was observed. A single species pair with the nearest neighbor K2P distance of less than 1% was observed for *Jaydia smithi* Kotthaus, 1970 and *Jaydia truncata* (Bleeker, 1855), however, reciprocal monophyly was observed.

**TABLE 2:** Summary statistics of pairwise K2P genetic distances (%) among sequences within species, among species within genus, and among species within family.

Level	*n*	Min dist	Mean dist	Max dist	SE dist
Within species	285	0.00	0.0036	0.0864	0.0004
Within genus	185	0.00	0.0696	0.28	0.0027
Within family	88	0.00	0.15	0.3398	0.0046

**FIGURE 5:**
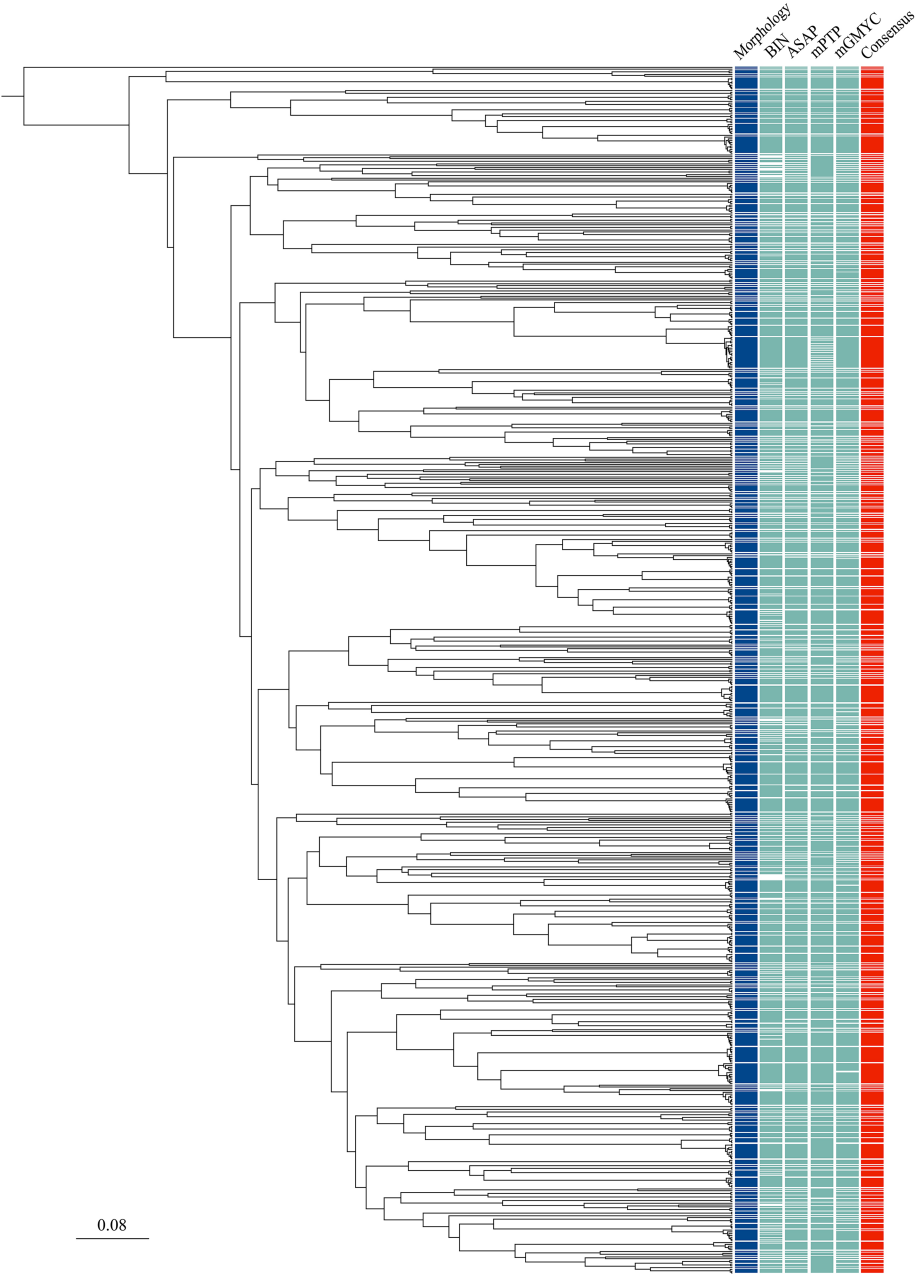
Bayesian ultrametric tree of the 1080 COI sequences including species delimitation using morphological characters, and DNA‐based species delimitation schemes derived from BIN, ASAP, mPTP, and mGMYC with final delimitation schemes based on majority rule consensus among the five methods (red).

The number of MOTUs delimited was 279, 284, 294, and 211 for RESL, ASAP, mGMYC, and mPTP, respectively (Figure 5; Table S4). The number of BINs was underestimated due to the absence of BIN numbers for 16 sequences belonging to 11 species in BOLD. The consensus delimitation scheme based on a majority rule resulted in 285 MOTUs among 285 species, and a comparison of the two revealed some differences between MOTUs and species boundaries (Figure 4). Plots of maximum intraspecific and nearest neighbor K2P distances for MOTUs indicate a wider barcode gap (Figure 4a,b). Among the three species with a maximum intraspecific K2P genetic distance above 2%, only *Scomberomorus guttatus* display more than a single MOTU (Table 3). However, the two MOTU detected within *S. guttatus* present both an elongated body silvery‐white ventrally, a lateral line gradually curving down toward caudal peduncle, numerous small black spots on side of body, a first dorsal‐fin black until the eighth fin spine, and an intestine with 2 folds and 3 limbs, a character which distinguishes *S. guttatus* from all other *Scomberomorus* species (Figure S2). A single case of MOTU shared by more than one species was observed with the two *Jaydia* species (Table 3), but no haplotype sharing was observed and the two species were distinguished by their coloration patterns. *Jaydia truncata* display a distinct dark stripe along the outer edge of the caudal fin which is absent or thinner in *J. smithi* and *J. smithi* display a white stripe below the dark spot of first dorsal fin which is absent in *J. truncate* (Figure S3).

**TABLE 3:** Summary statistics of genetic K2P distances for species displaying a mismatch between MOTUs and morphological delimitations, including species with more than a single MOTUs, and MOTUs encompassing more than a single species.

Species/MOTUs	Max. Intraspecific distance	Nearest neighbor distance
Species comprising two MOTUs		
*Scomberomorus guttatus*	0.0864	0.1251
OTU61 (BIN: not available)	–	0.0873
OTU266 (BIN: AAB0202)	–	0.0873
Pairs of species sharing one MOTU		
*Jaydia smithi*	0.0015	0.0077
*Jaydia truncata*	0.0015	0.0077
OTU197	0.0096	0.1169

## 4 DISCUSSION

Since its earliest inception (Hebert, Cywinska, et al., 2003), DNA barcoding has been increasingly used to standardize molecular methods of species identification (Blagoev et al., 2015; Hebert et al., 2013) and accelerated the pace of species discovery by promoting the use of DNA sequences in biodiversity inventories (Arida et al., 2021; Delrieu‐Trottin et al., 2019; Shan et al., 2021; Sonet et al., 2019). The development of DNA barcode reference libraries provides valuable genetic resources to help out pinpoint species of concern such as endangered or data‐deficient species by providing accurate identifications and as such provide scientific tools and knowledge for the sustainable management of fishing resources in regional fisheries. Previous large‐scale DNA barcoding campaign usually reported a barcode gap in 90% of the species examined, with the 10% left representing cases of recent divergence and/or introgressive hybridization (April et al., 2011; Delrieu‐Trottin et al., 2019). In this study, however, a barcode gap was detected in the 285 species of the study and well above 90%.

The nearest neighbor genetic distance was 40.43‐fold higher on average than the maximum intraspecific genetic distance. The high ratio recovered here indicates that species consist of tight clusters of DNA barcodes. This ratio may decrease, however, if expanding spatial scale and/or taxonomic coverage as it may result in increased maximum intraspecific genetic distances (Bergsten et al., 2012; Chen et al., 2022) and incorporating more closely related species (Dahruddin et al., 2017; Hubert et al., 2012). Comparisons with other studies confirm the geographic scale effect as the average intraspecific genetic distance estimated here (0.36%) is lower than observed elsewhere at wider spatial scales such as the Mediterranean (0.39%), Australian shores (0.39%) and the Indo‐Pacific (>1%) (Hubert et al., 2012, 2017; Landi et al., 2014; Ward et al., 2005). However, with a value of 6.96%, the average genetic distances among congeneric species observed here are lower than estimated for Mediterranean fishes (8.91%), Australian fishes (9.93%), or Indo‐Pacific fishes (>14%) (Hubert et al., 2012, 2017; Landi et al., 2014; Ward et al., 2005; Xu et al., 2021). This trend suggests that the Beibu Gulf hosts closer related species than other DNA‐barcoded region in the Pacific. These differences between genera perhaps reflect the average age of congeneric species divergence, as some species are younger than others within genera, as well as some genera relative to each other (Ward et al., 2005).

This trend is exemplified by the *Jaydia* species pair (Apogonidae), whose species delimitation method failed to separate and group in a single MOTU due to their close genetic affinity, despite several diagnostic morphological characters distinguishing them including caudal and dorsal fin coloration patterns. However, this trend is common among Apogonidae species (Luo et al., 2021; Mabuchi et al., 2014; Yi et al., 2021). Small genetic distances and a relatively young timeframe of diversification are likely to account for the lack of discrimination by mPTP of several MOTUs. By contrast, exceptions to the low intraspecific genetic distance and general match between species and MOTUs were detected. Cases of cryptic diversity are observed in less than 1% of the species under scrutiny with 2 MOTUs delineated within *Scomberomorus guttatus* with a maximum genetic distance of 0.0864 between the two MOTUs. This proportion (1%) is strikingly similar to previous assessments at regional scale in the South China Sea where 1.7% of the species contained cryptic and highly divergent lineages (Xu et al., 2021). Cases of cryptic diversity were limited, however, cryptic diversity in marine fishes usually occurs on much larger geographical scale in oceans than rivers because of higher connectivity in the marine realm (Hubert et al., 2012, 2017).

Previous studies on fish eggs in the area revealed that the families Carangidae and Scianidae are the most abundant and diverse in the pelagic environments of the gulf (Hou et al., 2022). The present study was in agreement with these observations as the family Carangidae is the most diverse with the highest number of species collected here (Figure 3, Table 1). We also observed that the family Malacanthidae is predominately spread in the middle region while the family Engraulidae is predominately located in the northeast region (Table S1). Historical records based on deep trawling identified *Acropoma japonicum* Günther, 1859 and *Photopectoralis bindus* (Valenciennes, 1835) as the dominant species in deep waters of the Beibu Gulf (Hou et al., 2022; Sun et al., 2019; Wang et al., 2011), two species that were captured at multiple sites during the trawling sessions conducted here (Table S1). This study shows that the most species‐rich groups in oceanic trawling are only rarely those targeted by fisheries as reflected by the most abundant families in fish stalls (Figure 3) which largely differ from the trawling capture. Of the top 10 families in terms of species richness from trawling only seven are in the top 10 families in terms of species richness at fish stalls (Lutjanidae, Clupeidae, Sciaenidae, Serranidae, Engraulidae, Nemipteridae, Scorpaenidae), however, the diversity of species we collected at fish stalls for these families is lower than that of previous studies (Sun et al., 2010; Wang et al., 2011), an observation in agreement with the general trend of stock collapses previously reported in the area during the last decades (Yuan et al., 2011; Zhang et al., 2021).

The checklist of fish species present in the Beibu Gulf, established by Sun et al. (2010), lists 626 species distributed in 370 genera, 140 families and 29 orders (Table 1), that is, a number of species twice as high as that reported by more recent fisheries and ichthyological studies between 2011 and 2021, and which mention between 152 and 301 species in the Beibu Gulf (Yuan et al., 2011; Zhang et al., 2021). The ichthyodiversity reported here is consistent with the most recent estimates of the fish species richness in the Beibu Gulf with 263 species collected during our study. These large discrepancies between historical and recent ichthyological surveys, repeatedly reported during the last decades and observed here as well, might be explained by several aspects. First, the sampling strategy implemented here based on deep trawling with medium size mesh and, completed by visiting fish stalls on a regular basis may account for the absence of several orders such as Syngnathiformes which mostly correspond to small‐size species that are not captured with nets of 50 mm mesh size and are not targeted by commercial fisheries. Taxonomic gaps in our sampling were also suggested by the species accumulation curve for the sampled species during the present study as a plateau is not reached, that is, taxonomic coverage can be improved. Second, taxonomic uncertainties also can be a substantial source of discrepancies among studies and result in inflated number of species in historical records due to misidentifications (Dahruddin et al., 2017), a trend that likely explains much of the differences observed here compared to historical records (Table 1). Third, the lack of observation here of several orders, such as Rajiformes (rays), despite a multi‐annual sampling, is surprising as rays are usually captured by bottom trawling and used to be targeted by fisheries in the past. This result further questions the conservation status of many commercially exploited species in the Beibu Gulf and is consistent with previous analyses of trawl data from the main economic fish species from the 1960s to 2002 which evidenced that some dominant species, such as *Lutjanus sanguineus* (Cuvier, 1828), *Terapon theraps* Cuvier, 1829, *Gerres filamentosus* Cuvier, 1829, *Carcharhinus menisorrah* (Bibron, 1839) and *Gymnocranius griseus* (Temminck & Schlegel, 1843) have experienced serious declines (Wang et al., 2011; Yuan et al., 2011; Zhang et al., 2021). This trend of stock collapse is further corroborated by the observation of multiple replacement of high‐value species (*L. sanguineus*, *T. theraps*, *G. filamentosus*, *G. griseus*, and so on) by medium and low commercial value species such as *A. japonicum* and several species of the family Leiognathidae (Wang et al., 2011; Yuan et al., 2011; Zhang et al., 2021). Even if the study of the stocks was not the initial goal of the present study, the observations provided here are in agreement with the depletion of the marine resources in the Beibu Gulf and are of great concern for the sustainability of the fisheries and food security in the near future.

## 5 CONCLUSION

With 285 species, this study provides the first large‐scale DNA barcode reference library of the fishes of the Beibu Gulf, with a particular emphasis on commercially exploited species. The close match between species and MOTUs supports the efficiency of DNA barcoding in the context of the fish fauna of the Beibu Gulf and opens up new perspectives for the monitoring and management of Beibu Gulf fisheries by enabling automated species identification and next‐generation monitoring of the 285 species referenced here. However, this study warrants further ichthyological exploration of the Beibu Gulf using DNA barcodes as major differences between the number of species sampled during the course of the present study and the literature, as well as unsaturated accumulation curves, suggest that a substantial portion of the Beibu Gulf ichthyofauna awaits being referenced. Although these discrepancies may originate from multiple factors, the replacement of several high‐value species by medium to low‐value species at fish stalls suggest that stock collapse might also be responsible for these large differences. In the context of the decline of the fisheries in the Beibu Gulf, we provide a comprehensive DNA barcode reference library, which will be of convenient use in fisheries management and relevant scientific research.

## AUTHOR CONTRIBUTIONS

**Changping Jiang:** Conceptualization (equal); data curation (equal); formal analysis (equal); methodology (equal); writing – original draft (equal). **Murong Yi:** Data curation (equal); formal analysis (equal); methodology (equal); writing – original draft (equal). **Zhisen Luo:** Formal analysis (supporting); investigation (supporting); validation (supporting); writing – review and editing (supporting). **Xiongbo He:** Formal analysis (supporting); investigation (supporting); validation (supporting); writing – review and editing (supporting). **Hung‐Du Lin:** Formal analysis (supporting); investigation (supporting); validation (supporting); writing – review and editing (supporting). **Nicolas Hubert:** Formal analysis (supporting); investigation (supporting); methodology (supporting); supervision (supporting); validation (supporting); writing – original draft (supporting); writing – review and editing (equal). **Yunrong Yan:** Conceptualization (lead); formal analysis (supporting); funding acquisition (lead); investigation (lead); project administration (lead); validation (lead); writing – original draft (equal); writing – review and editing (equal).

## CONFLICT OF INTEREST STATEMENT

The authors declare no competing interests.

## Supporting information


Figure S1.

Figure S2.

Figure S3.

Table S1.

Table S2.

Table S3.

Table S4.

## Data Availability

Sequence data are available on GenBank (accession numbers OQ552889–OQ553649, OQ559023–OQ559041, and OR523579–OR523587).
