# Supplementary figures and images for: DNA barcoding the ichthyofauna of the Beibu Gulf: Implications for fisheries management in a seafood market hub
Source: Ecol Evol. 2023 Dec 11;13(12):e10822. doi: 10.1002/ece3.10822 (PMC10711522; doi:10.1002/ece3.10822)

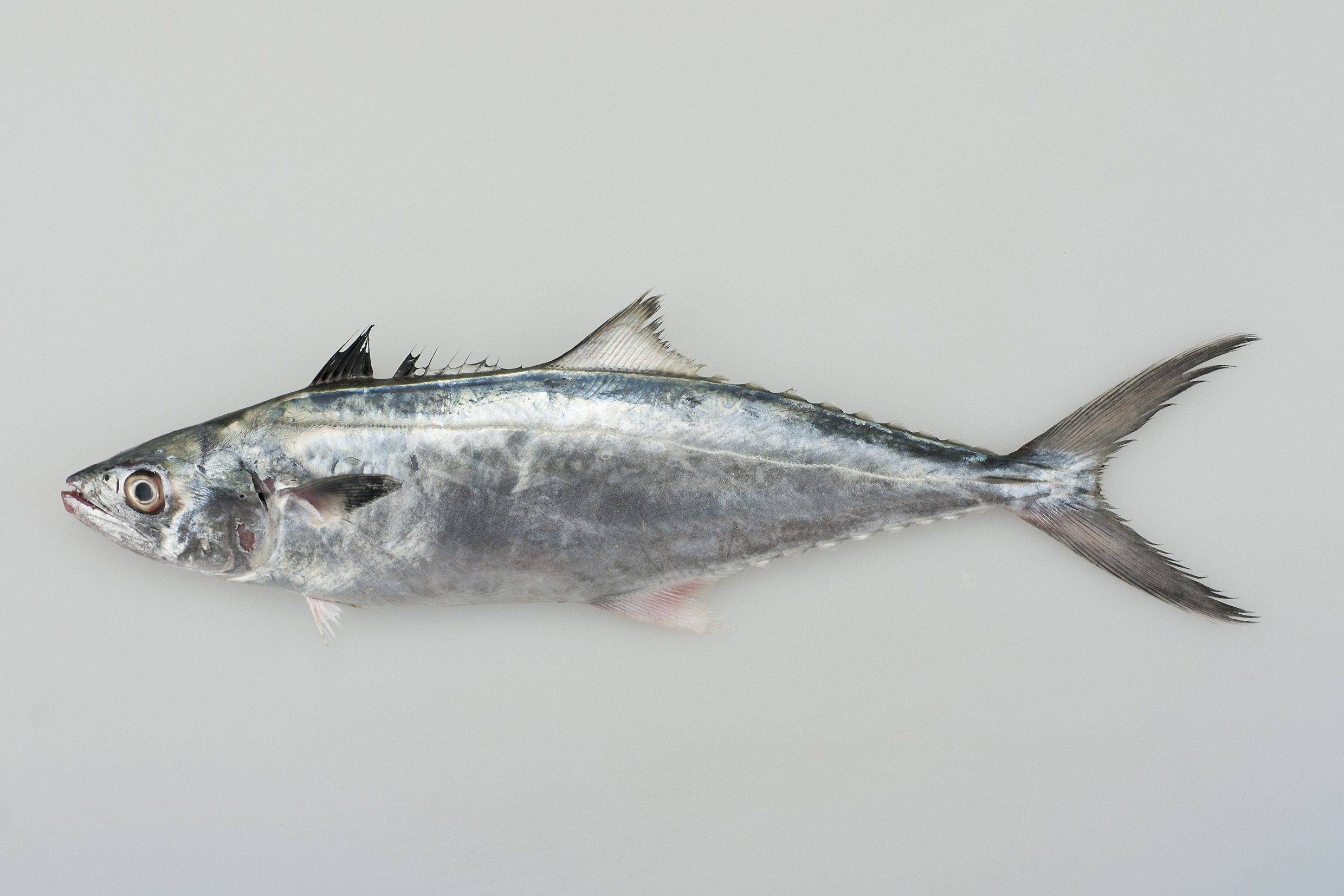

Supplement: Supplementary file 2 — Figure S2. [file ECE3-13-e10822-s007.jpg]

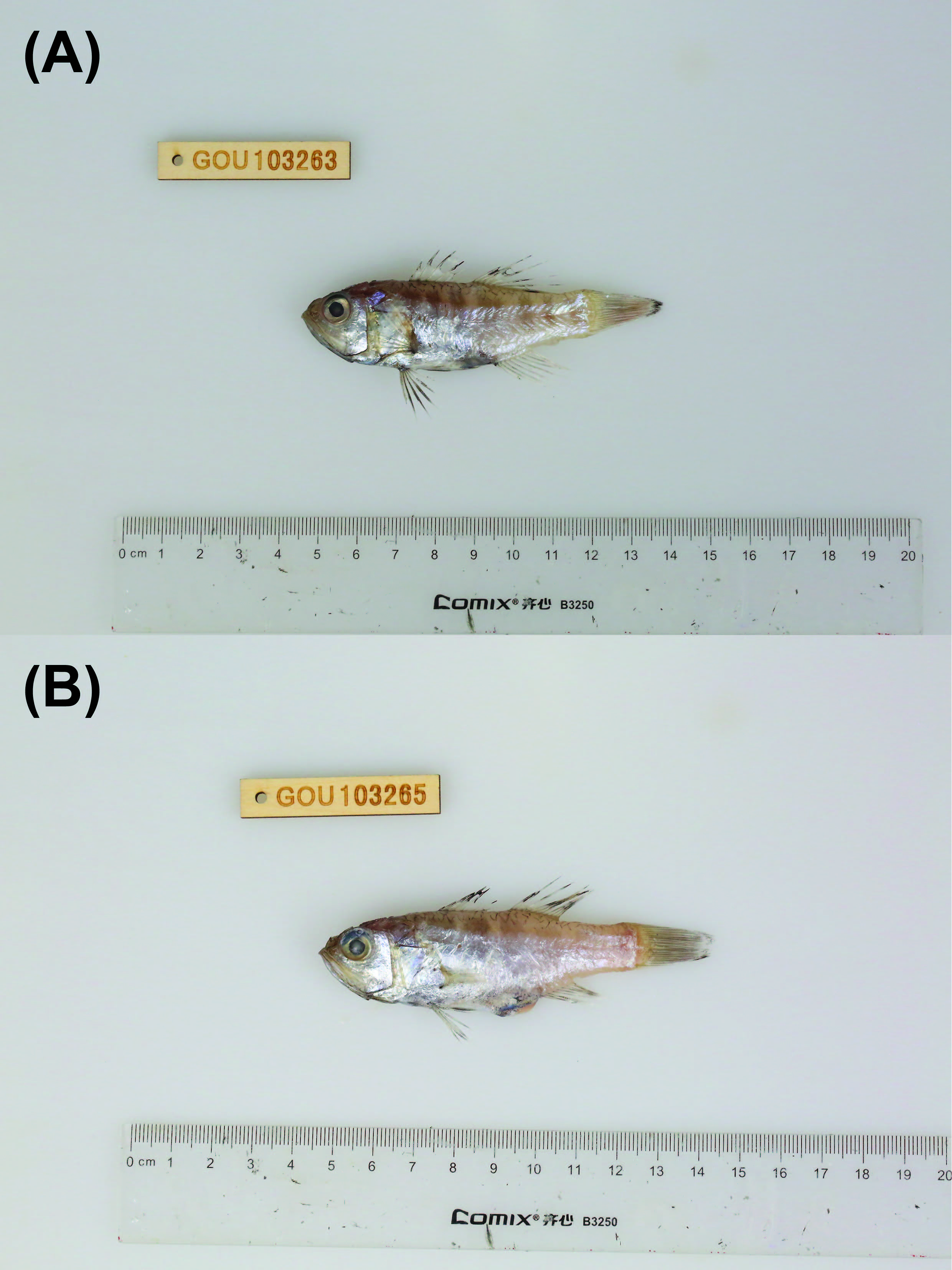

Supplement: Supplementary file 3 — Figure S3. [file ECE3-13-e10822-s004.jpg]
