# Supplementary material for: DNA barcoding the ichthyofauna of the Beibu Gulf: Implications for fisheries management in a seafood market hub
Source: Ecol Evol. 2023 Dec 11;13(12):e10822. doi: 10.1002/ece3.10822 (PMC10711522; doi:10.1002/ece3.10822)
Supplement: Supplementary file 1 — Figure S1. [file ECE3-13-e10822-s006.pdf]

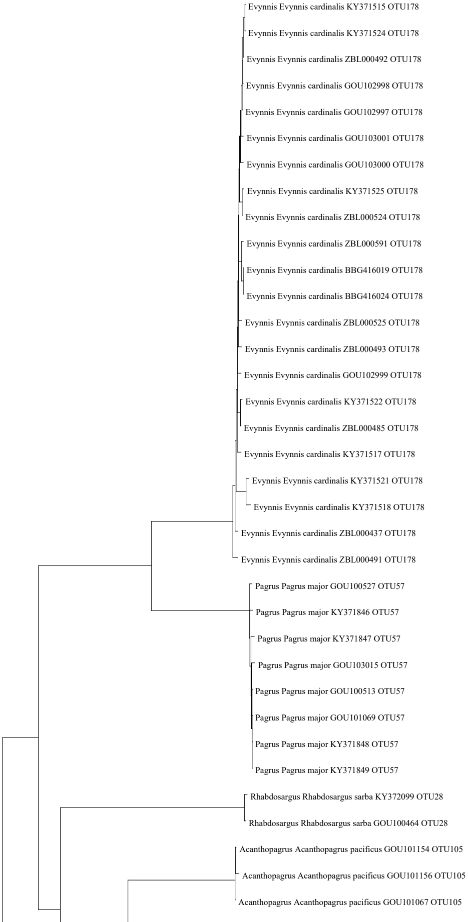
Evynnis Evynnis cardinalis KY371515 OTU178
Evynnis Evynnis cardinalis KY371524 OTU178
Evynnis Evynnis cardinalis ZBL000492 OTU178
Evynnis Evynnis cardinalis GOU102998 OTU178
Evynnis Evynnis cardinalis GOU102997 OTU178
Evynnis Evynnis cardinalis GOU103001 OTU178
Evynnis Evynnis cardinalis GOU103000 OTU178
Evynnis Evynnis cardinalis KY371525 OTU178
Evynnis Evynnis cardinalis ZBL000524 OTU178
Evynnis Evynnis cardinalis ZBL000591 OTU178
Evynnis Evynnis cardinalis BBG416019 OTU178
Evynnis Evynnis cardinalis BBG416024 OTU178
Evynnis Evynnis cardinalis ZBL000525 OTU178
Evynnis Evynnis cardinalis ZBL000493 OTU178
Evynnis Evynnis cardinalis GOU102999 OTU178
Evynnis Evynnis cardinalis KY371522 OTU178
Evynnis Evynnis cardinalis ZBL000485 OTU178
Evynnis Evynnis cardinalis KY371517 OTU178
Evynnis Evynnis cardinalis KY371521 OTU178
Evynnis Evynnis cardinalis KY371518 OTU178
Evynnis Evynnis cardinalis ZBL000437 OTU178
Evynnis Evynnis cardinalis ZBL000491 OTU178
Pagrus Pagrus major GOU100527 OTU57
Pagrus Pagrus major KY371846 OTU57
Pagrus Pagrus major KY371847 OTU57
Pagrus Pagrus major GOU103015 OTU57
Pagrus Pagrus major GOU100513 OTU57
Pagrus Pagrus major GOU101069 OTU57
Pagrus Pagrus major KY371848 OTU57
Pagrus Pagrus major KY371849 OTU57
Rhabdosargus Rhabdosargus sarba KY372099 OTU28
Rhabdosargus Rhabdosargus sarba GOU100464 OTU28
Acanthopagrus Acanthopagrus pacificus GOU101154 OTU105
Acanthopagrus Acanthopagrus pacificus GOU101156 OTU105
Acanthopagrus Acanthopagrus pacificus GOU101067 OTU105

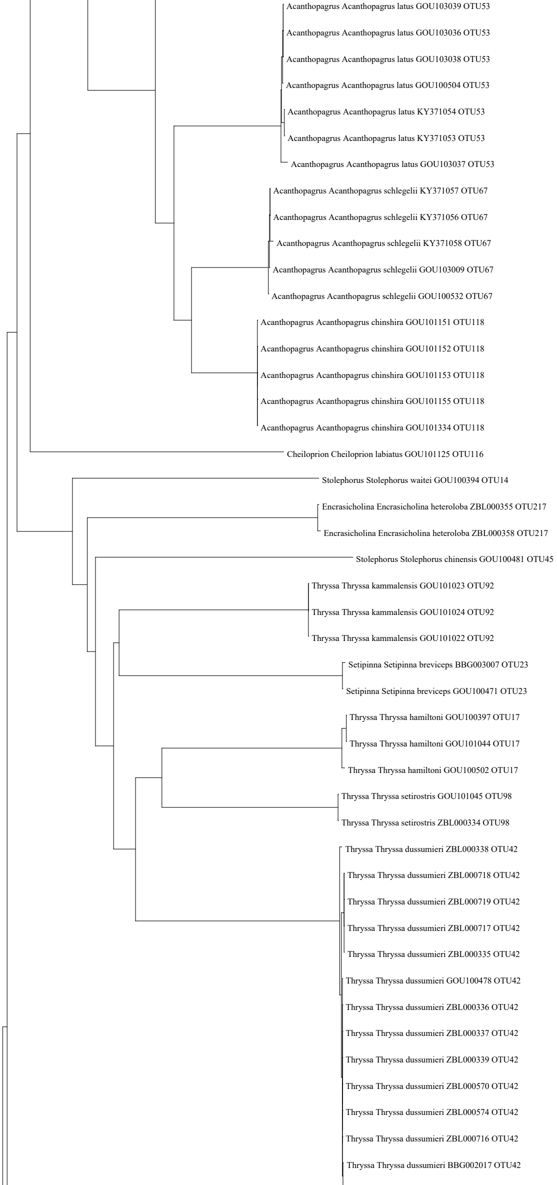

Acanthopagrus Acanthopagrus latus GOU103039 OTU53
Acanthopagrus Acanthopagrus latus GOU103036 OTU53
Acanthopagrus Acanthopagrus latus GOU103038 OTU53
Acanthopagrus Acanthopagrus latus GOU100504 OTU53
Acanthopagrus Acanthopagrus latus KY371054 OTU53
Acanthopagrus Acanthopagrus latus KY371053 OTU53
Acanthopagrus Acanthopagrus latus GOU103037 OTU53
Acanthopagrus Acanthopagrus schlegelii KY371057 OTU67
Acanthopagrus Acanthopagrus schlegelii KY371056 OTU67
Acanthopagrus Acanthopagrus schlegelii KY371058 OTU67
Acanthopagrus Acanthopagrus schlegelii GOU103009 OTU67
Acanthopagrus Acanthopagrus schlegelii GOU100532 OTU67
Acanthopagrus Acanthopagrus chinshira GOU101151 OTU118
Acanthopagrus Acanthopagrus chinshira GOU101152 OTU118
Acanthopagrus Acanthopagrus chinshira GOU101153 OTU118
Acanthopagrus Acanthopagrus chinshira GOU101155 OTU118
Acanthopagrus Acanthopagrus chinshira GOU101334 OTU118
Cheiloprion Cheiloprion labiatus GOU101125 OTU116
Stolephorus Stolephorus waitei GOU100394 OTU14
Encrasicholina Encrasicholina heteroloba ZBL000355 OTU217
Encrasicholina Encrasicholina heteroloba ZBL000358 OTU217
Stolephorus Stolephorus chinensis GOU100481 OTU45
Thryssa Thryssa kammalensis GOU101023 OTU92
Thryssa Thryssa kammalensis GOU101024 OTU92
Thryssa Thryssa kammalensis GOU101022 OTU92
Setipinna Setipinna breviceps BBG003007 OTU23
Setipinna Setipinna breviceps GOU100471 OTU23
Thryssa Thryssa hamiltoni GOU100397 OTU17
Thryssa Thryssa hamiltoni GOU101044 OTU17
Thryssa Thryssa hamiltoni GOU100502 OTU17
Thryssa Thryssa setirostris GOU101045 OTU98
Thryssa Thryssa setirostris ZBL000334 OTU98
Thryssa Thryssa dussumieri ZBL000338 OTU42
Thryssa Thryssa dussumieri ZBL000718 OTU42
Thryssa Thryssa dussumieri ZBL000719 OTU42
Thryssa Thryssa dussumieri ZBL000717 OTU42
Thryssa Thryssa dussumieri ZBL000335 OTU42
Thryssa Thryssa dussumieri GOU100478 OTU42
Thryssa Thryssa dussumieri ZBL000336 OTU42
Thryssa Thryssa dussumieri ZBL000337 OTU42
Thryssa Thryssa dussumieri ZBL000339 OTU42
Thryssa Thryssa dussumieri ZBL000570 OTU42
Thryssa Thryssa dussumieri ZBL000574 OTU42
Thryssa Thryssa dussumieri ZBL000716 OTU42
Thryssa Thryssa dussumieri BBG002017 OTU42

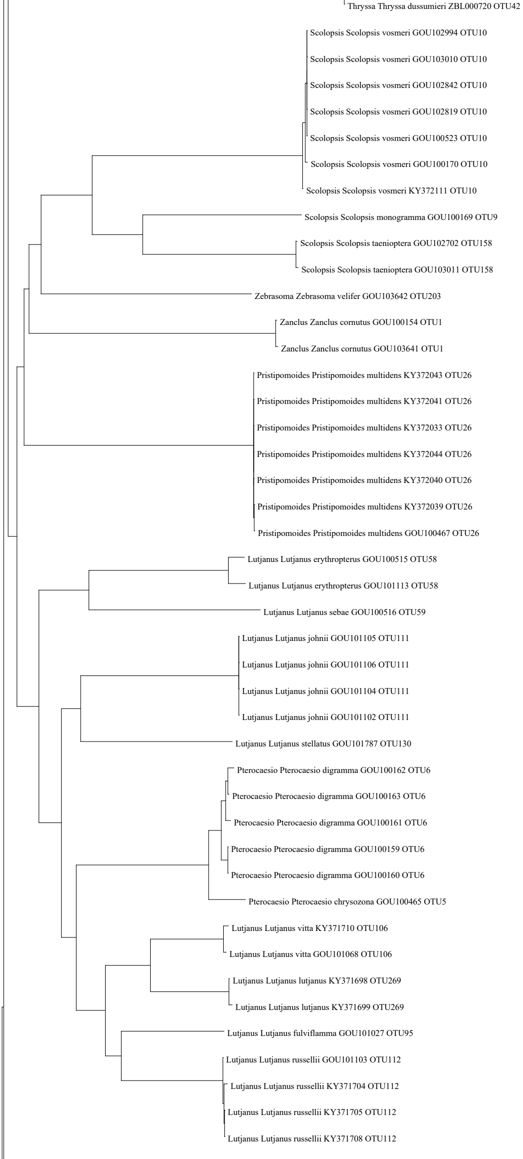
Thryssa Thryssa dussumieri ZBL000720 OTU42
Scolopsis Scolopsis vosmeri GOU102994 OTU10
Scolopsis Scolopsis vosmeri GOU103010 OTU10
Scolopsis Scolopsis vosmeri GOU102842 OTU10
Scolopsis Scolopsis vosmeri GOU102819 OTU10
Scolopsis Scolopsis vosmeri GOU100523 OTU10
Scolopsis Scolopsis vosmeri GOU100170 OTU10
Scolopsis Scolopsis vosmeri KY372111 OTU10
Scolopsis Scolopsis monogramma GOU100169 OTU9
Scolopsis Scolopsis taenioptera GOU102702 OTU158
Scolopsis Scolopsis taenioptera GOU103011 OTU158
Zebrasoma Zebrasoma velifer GOU103642 OTU203
Zanclus Zanclus cornutus GOU100154 OTU1
Zanclus Zanclus cornutus GOU103641 OTU1
Pristipomoides Pristipomoides multidens KY372043 OTU26
Pristipomoides Pristipomoides multidens KY372041 OTU26
Pristipomoides Pristipomoides multidens KY372033 OTU26
Pristipomoides Pristipomoides multidens KY372044 OTU26
Pristipomoides Pristipomoides multidens KY372040 OTU26
Pristipomoides Pristipomoides multidens KY372039 OTU26
Pristipomoides Pristipomoides multidens GOU100467 OTU26
Lutjanus Lutjanus erythropterus GOU100515 OTU58
Lutjanus Lutjanus erythropterus GOU101113 OTU58
Lutjanus Lutjanus sebae GOU100516 OTU59
Lutjanus Lutjanus johnii GOU101105 OTU111
Lutjanus Lutjanus johnii GOU101106 OTU111
Lutjanus Lutjanus johnii GOU101104 OTU111
Lutjanus Lutjanus johnii GOU101102 OTU111
Lutjanus Lutjanus stellatus GOU101787 OTU130
Pterocaesio Pterocaesio digramma GOU100162 OTU6
Pterocaesio Pterocaesio digramma GOU100163 OTU6
Pterocaesio Pterocaesio digramma GOU100161 OTU6
Pterocaesio Pterocaesio digramma GOU100159 OTU6
Pterocaesio Pterocaesio digramma GOU100160 OTU6
Pterocaesio Pterocaesio chrysozona GOU100465 OTU5
Lutjanus Lutjanus vitta KY371710 OTU106
Lutjanus Lutjanus vitta GOU101068 OTU106
Lutjanus Lutjanus lutjanus KY371698 OTU269
Lutjanus Lutjanus lutjanus KY371699 OTU269
Lutjanus Lutjanus fulviflamma GOU101027 OTU95
Lutjanus Lutjanus russellii GOU101103 OTU112
Lutjanus Lutjanus russellii KY371704 OTU112
Lutjanus Lutjanus russellii KY371705 OTU112
Lutjanus Lutjanus russellii KY371708 OTU112

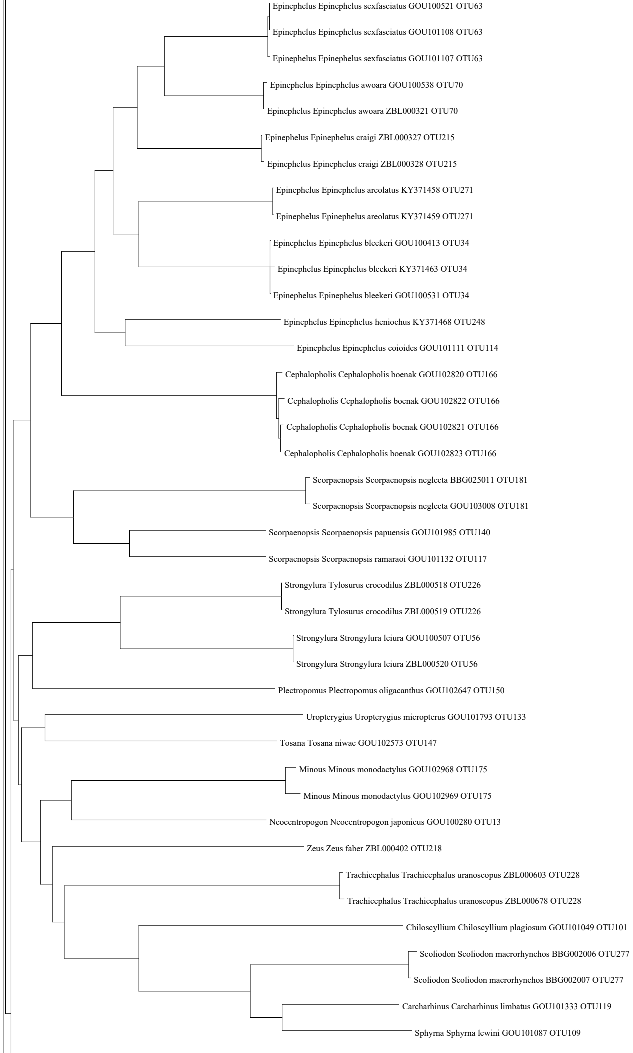
Epinephelus Epinephelus sexfasciatus GOU100521 OTU63
Epinephelus Epinephelus sexfasciatus GOU101108 OTU63
Epinephelus Epinephelus sexfasciatus GOU101107 OTU63
Epinephelus Epinephelus awoara GOU100538 OTU70
Epinephelus Epinephelus awoara ZBL000321 OTU70
Epinephelus Epinephelus craigi ZBL000327 OTU215
Epinephelus Epinephelus craigi ZBL000328 OTU215
Epinephelus Epinephelus areolatus KY371458 OTU271
Epinephelus Epinephelus areolatus KY371459 OTU271
Epinephelus Epinephelus bleekeri GOU100413 OTU34
Epinephelus Epinephelus bleekeri KY371463 OTU34
Epinephelus Epinephelus bleekeri GOU100531 OTU34
Epinephelus Epinephelus heniochus KY371468 OTU248
Epinephelus Epinephelus coioides GOU101111 OTU114
Cephalopholis Cephalopholis boenak GOU102820 OTU166
Cephalopholis Cephalopholis boenak GOU102822 OTU166
Cephalopholis Cephalopholis boenak GOU102821 OTU166
Cephalopholis Cephalopholis boenak GOU102823 OTU166
Scorpaenopsis Scorpaenopsis neglecta BBG025011 OTU181
Scorpaenopsis Scorpaenopsis neglecta GOU103008 OTU181
Scorpaenopsis Scorpaenopsis papuensis GOU101985 OTU140
Scorpaenopsis Scorpaenopsis ramaraoi GOU101132 OTU117
Strongylura Tylosurus crocodilus ZBL000518 OTU226
Strongylura Tylosurus crocodilus ZBL000519 OTU226
Strongylura Strongylura leiura GOU100507 OTU56
Strongylura Strongylura leiura ZBL000520 OTU56
Plectropomus Plectropomus oligacanthus GOU102647 OTU150
Uropterygius Uropterygius micropterus GOU101793 OTU133
Tosana Tosana niwae GOU102573 OTU147
Minous Minous monodactylus GOU102968 OTU175
Minous Minous monodactylus GOU102969 OTU175
Neocentropogon Neocentropogon japonicus GOU100280 OTU13
Zeus Zeus faber ZBL000402 OTU218
Trachicephalus Trachicephalus uranoscopus ZBL000603 OTU228
Trachicephalus Trachicephalus uranoscopus ZBL000678 OTU228
Chiloscyllium Chiloscyllium plagiosum GOU101049 OTU101
Scoliodon Scoliodon macrorhynchos BBG002006 OTU277
Scoliodon Scoliodon macrorhynchos BBG002007 OTU277
Carcharhinus Carcharhinus limbatus GOU101333 OTU119
Sphyrna Sphyrna lewini GOU101087 OTU109

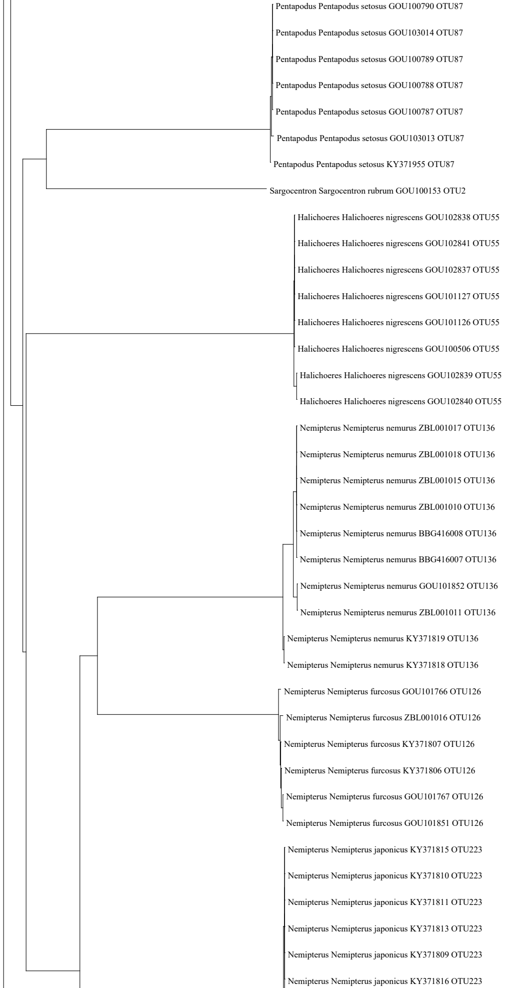
Pentapodus Pentapodus setosus GOU100790 OTU87
Pentapodus Pentapodus setosus GOU103014 OTU87
Pentapodus Pentapodus setosus GOU100789 OTU87
Pentapodus Pentapodus setosus GOU100788 OTU87
Pentapodus Pentapodus setosus GOU100787 OTU87
Pentapodus Pentapodus setosus GOU103013 OTU87
Pentapodus Pentapodus setosus KY371955 OTU87
Sargocentron Sargocentron rubrum GOU100153 OTU2
Halichoeres Halichoeres nigrescens GOU102838 OTU55
Halichoeres Halichoeres nigrescens GOU102841 OTU55
Halichoeres Halichoeres nigrescens GOU102837 OTU55
Halichoeres Halichoeres nigrescens GOU101127 OTU55
Halichoeres Halichoeres nigrescens GOU101126 OTU55
Halichoeres Halichoeres nigrescens GOU100506 OTU55
Halichoeres Halichoeres nigrescens GOU102839 OTU55
Halichoeres Halichoeres nigrescens GOU102840 OTU55
Nemipterus Nemipterus nemurus ZBL001017 OTU136
Nemipterus Nemipterus nemurus ZBL001018 OTU136
Nemipterus Nemipterus nemurus ZBL001015 OTU136
Nemipterus Nemipterus nemurus ZBL001010 OTU136
Nemipterus Nemipterus nemurus BBG416008 OTU136
Nemipterus Nemipterus nemurus BBG416007 OTU136
Nemipterus Nemipterus nemurus GOU101852 OTU136
Nemipterus Nemipterus nemurus ZBL001011 OTU136
Nemipterus Nemipterus nemurus KY371819 OTU136
Nemipterus Nemipterus nemurus KY371818 OTU136
Nemipterus Nemipterus furcosus GOU101766 OTU126
Nemipterus Nemipterus furcosus ZBL001016 OTU126
Nemipterus Nemipterus furcosus KY371807 OTU126
Nemipterus Nemipterus furcosus KY371806 OTU126
Nemipterus Nemipterus furcosus GOU101767 OTU126
Nemipterus Nemipterus furcosus GOU101851 OTU126
Nemipterus Nemipterus japonicus KY371815 OTU223
Nemipterus Nemipterus japonicus KY371810 OTU223
Nemipterus Nemipterus japonicus KY371811 OTU223
Nemipterus Nemipterus japonicus KY371813 OTU223
Nemipterus Nemipterus japonicus KY371809 OTU223
Nemipterus Nemipterus japonicus KY371816 OTU223

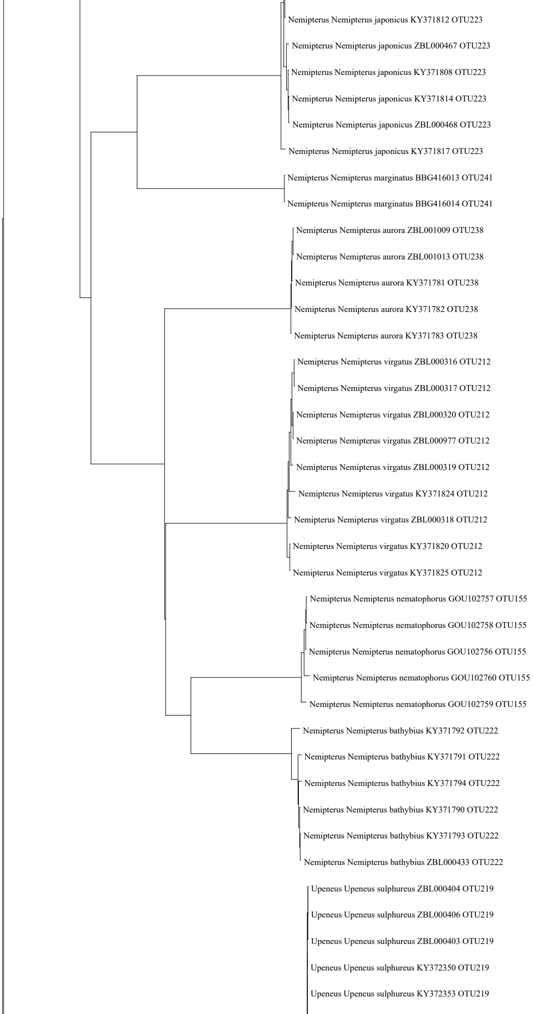
Nemipterus Nemipterus japonicus KY371812 OTU223
Nemipterus Nemipterus japonicus ZBL000467 OTU223
Nemipterus Nemipterus japonicus KY371808 OTU223
Nemipterus Nemipterus japonicus KY371814 OTU223
Nemipterus Nemipterus japonicus ZBL000468 OTU223
Nemipterus Nemipterus japonicus KY371817 OTU223
Nemipterus Nemipterus marginatus BBG416013 OTU241
Nemipterus Nemipterus marginatus BBG416014 OTU241
Nemipterus Nemipterus aurora ZBL001009 OTU238
Nemipterus Nemipterus aurora ZBL001013 OTU238
Nemipterus Nemipterus aurora KY371781 OTU238
Nemipterus Nemipterus aurora KY371782 OTU238
Nemipterus Nemipterus aurora KY371783 OTU238
Nemipterus Nemipterus virgatus ZBL000316 OTU212
Nemipterus Nemipterus virgatus ZBL000317 OTU212
Nemipterus Nemipterus virgatus ZBL000320 OTU212
Nemipterus Nemipterus virgatus ZBL000977 OTU212
Nemipterus Nemipterus virgatus ZBL000319 OTU212
Nemipterus Nemipterus virgatus KY371824 OTU212
Nemipterus Nemipterus virgatus ZBL000318 OTU212
Nemipterus Nemipterus virgatus KY371820 OTU212
Nemipterus Nemipterus virgatus KY371825 OTU212
Nemipterus Nemipterus nematophorus GOU102757 OTU155
Nemipterus Nemipterus nematophorus GOU102758 OTU155
Nemipterus Nemipterus nematophorus GOU102756 OTU155
Nemipterus Nemipterus nematophorus GOU102760 OTU155
Nemipterus Nemipterus nematophorus GOU102759 OTU155
Nemipterus Nemipterus bathybius KY371792 OTU222
Nemipterus Nemipterus bathybius KY371791 OTU222
Nemipterus Nemipterus bathybius KY371794 OTU222
Nemipterus Nemipterus bathybius KY371790 OTU222
Nemipterus Nemipterus bathybius KY371793 OTU222
Nemipterus Nemipterus bathybius ZBL000433 OTU222
Upeneus Upeneus sulphureus ZBL000404 OTU219
Upeneus Upeneus sulphureus ZBL000406 OTU219
Upeneus Upeneus sulphureus ZBL000403 OTU219
Upeneus Upeneus sulphureus KY372350 OTU219
Upeneus Upeneus sulphureus KY372353 OTU219

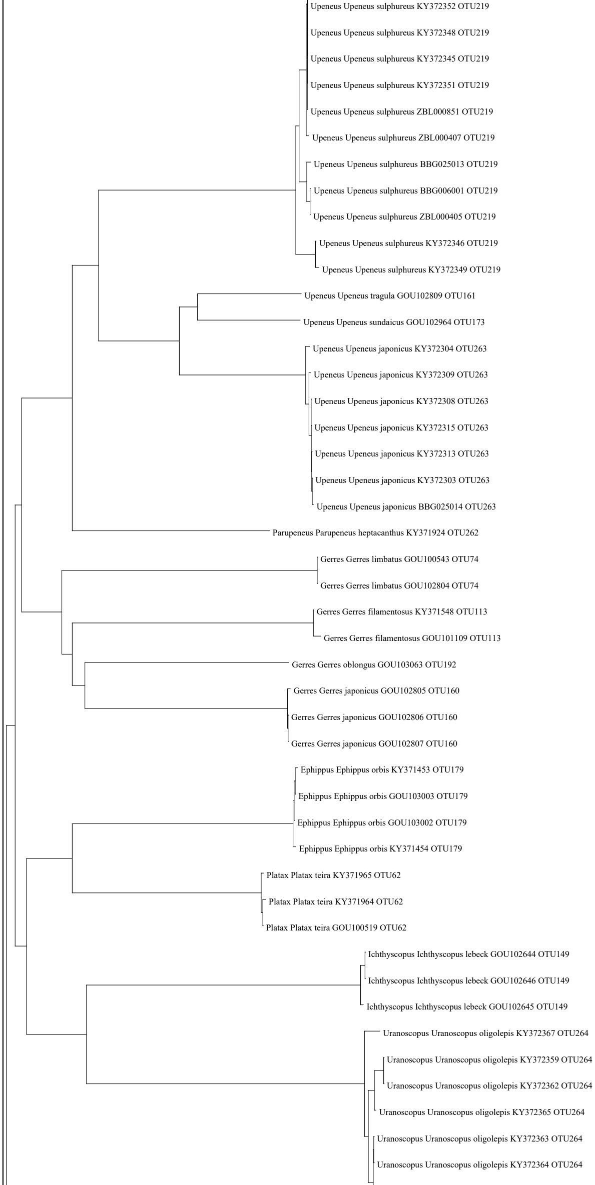
Upeneus Upeneus sulphureus KY372352 OTU219
Upeneus Upeneus sulphureus KY372348 OTU219
Upeneus Upeneus sulphureus KY372345 OTU219
Upeneus Upeneus sulphureus KY372351 OTU219
Upeneus Upeneus sulphureus ZBL000851 OTU219
Upeneus Upeneus sulphureus ZBL000407 OTU219
Upeneus Upeneus sulphureus BBG025013 OTU219
Upeneus Upeneus sulphureus BBG006001 OTU219
Upeneus Upeneus sulphureus ZBL000405 OTU219
Upeneus Upeneus sulphureus KY372346 OTU219
Upeneus Upeneus sulphureus KY372349 OTU219
Upeneus Upeneus tragula GOU102809 OTU161
Upeneus Upeneus sundaicus GOU102964 OTU173
Upeneus Upeneus japonicus KY372304 OTU263
Upeneus Upeneus japonicus KY372309 OTU263
Upeneus Upeneus japonicus KY372308 OTU263
Upeneus Upeneus japonicus KY372315 OTU263
Upeneus Upeneus japonicus KY372313 OTU263
Upeneus Upeneus japonicus KY372303 OTU263
Upeneus Upeneus japonicus BBG025014 OTU263
Parupeneus Parupeneus heptacanthus KY371924 OTU262
Gerres Gerres limbatus GOU100543 OTU74
Gerres Gerres limbatus GOU102804 OTU74
Gerres Gerres filamentosus KY371548 OTU113
Gerres Gerres filamentosus GOU101109 OTU113
Gerres Gerres oblongus GOU103063 OTU192
Gerres Gerres japonicus GOU102805 OTU160
Gerres Gerres japonicus GOU102806 OTU160
Gerres Gerres japonicus GOU102807 OTU160
Ephippus Ephippus orbis KY371453 OTU179
Ephippus Ephippus orbis GOU103003 OTU179
Ephippus Ephippus orbis GOU103002 OTU179
Ephippus Ephippus orbis KY371454 OTU179
Platax Platax teira KY371965 OTU62
Platax Platax teira KY371964 OTU62
Platax Platax teira GOU100519 OTU62
Ichthyscopus Ichthyscopus lebeck GOU102644 OTU149
Ichthyscopus Ichthyscopus lebeck GOU102646 OTU149
Ichthyscopus Ichthyscopus lebeck GOU102645 OTU149
Uranoscopus Uranoscopus oligolepis KY372367 OTU264
Uranoscopus Uranoscopus oligolepis KY372359 OTU264
Uranoscopus Uranoscopus oligolepis KY372362 OTU264
Uranoscopus Uranoscopus oligolepis KY372365 OTU264
Uranoscopus Uranoscopus oligolepis KY372363 OTU264
Uranoscopus Uranoscopus oligolepis KY372364 OTU264

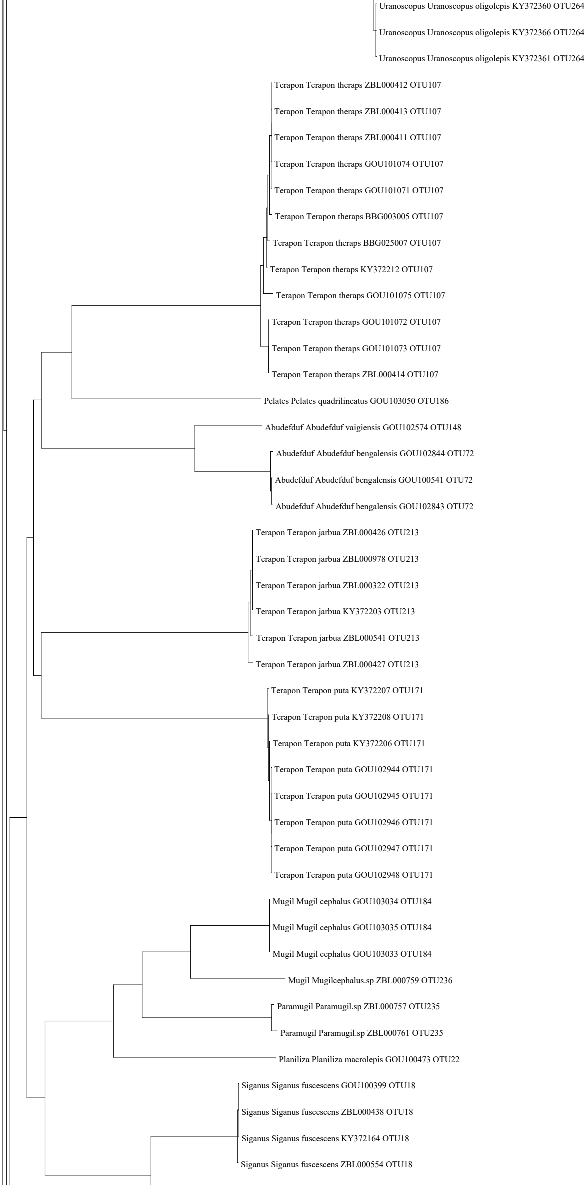
Uranoscopus Uranoscopus oligolepis KY372360 OTU264
Uranoscopus Uranoscopus oligolepis KY372366 OTU264
Uranoscopus Uranoscopus oligolepis KY372361 OTU264
Terapon Terapon theraps ZBL000412 OTU107
Terapon Terapon theraps ZBL000413 OTU107
Terapon Terapon theraps ZBL000411 OTU107
Terapon Terapon theraps GOU101074 OTU107
Terapon Terapon theraps GOU101071 OTU107
Terapon Terapon theraps BBG003005 OTU107
Terapon Terapon theraps BBG025007 OTU107
Terapon Terapon theraps KY372212 OTU107
Terapon Terapon theraps GOU101075 OTU107
Terapon Terapon theraps GOU101072 OTU107
Terapon Terapon theraps GOU101073 OTU107
Terapon Terapon theraps ZBL000414 OTU107
Pelates Pelates quadrilineatus GOU103050 OTU186
Abudefduf Abudefduf vaigiensis GOU102574 OTU148
Abudefduf Abudefduf bengalensis GOU102844 OTU72
Abudefduf Abudefduf bengalensis GOU100541 OTU72
Abudefduf Abudefduf bengalensis GOU102843 OTU72
Terapon Terapon jarbua ZBL000426 OTU213
Terapon Terapon jarbua ZBL000978 OTU213
Terapon Terapon jarbua ZBL000322 OTU213
Terapon Terapon jarbua KY372203 OTU213
Terapon Terapon jarbua ZBL000541 OTU213
Terapon Terapon jarbua ZBL000427 OTU213
Terapon Terapon puta KY372207 OTU171
Terapon Terapon puta KY372208 OTU171
Terapon Terapon puta KY372206 OTU171
Terapon Terapon puta GOU102944 OTU171
Terapon Terapon puta GOU102945 OTU171
Terapon Terapon puta GOU102946 OTU171
Terapon Terapon puta GOU102947 OTU171
Terapon Terapon puta GOU102948 OTU171
Mugil Mugil cephalus GOU103034 OTU184
Mugil Mugil cephalus GOU103035 OTU184
Mugil Mugil cephalus GOU103033 OTU184
Mugil Mugilcephalus.sp ZBL000759 OTU236
Paramugil Paramugil.sp ZBL000757 OTU235
Paramugil Paramugil.sp ZBL000761 OTU235
Planiliza Planiliza macrolepis GOU100473 OTU22
Siganus Siganus fuscescens GOU100399 OTU18
Siganus Siganus fuscescens ZBL000438 OTU18
Siganus Siganus fuscescens KY372164 OTU18
Siganus Siganus fuscescens ZBL000554 OTU18

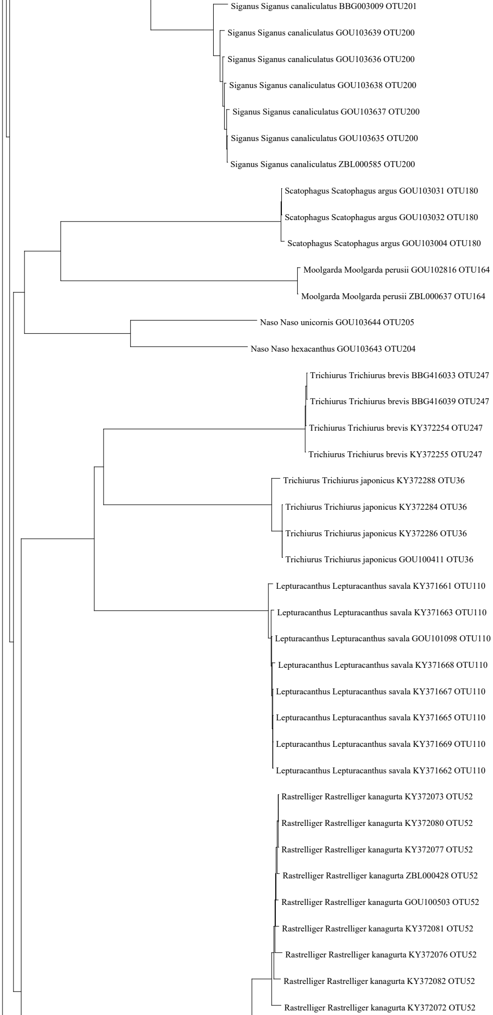
Siganus Siganus canaliculatus BBG003009 OTU201
Siganus Siganus canaliculatus GOU103639 OTU200
Siganus Siganus canaliculatus GOU103636 OTU200
Siganus Siganus canaliculatus GOU103638 OTU200
Siganus Siganus canaliculatus GOU103637 OTU200
Siganus Siganus canaliculatus GOU103635 OTU200
Siganus Siganus canaliculatus ZBL000585 OTU200
Scatophagus Scatophagus argus GOU103031 OTU180
Scatophagus Scatophagus argus GOU103032 OTU180
Scatophagus Scatophagus argus GOU103004 OTU180
Moolgarda Moolgarda perusii GOU102816 OTU164
Moolgarda Moolgarda perusii ZBL000637 OTU164
Naso Naso unicornis GOU103644 OTU205
Naso Naso hexacanthus GOU103643 OTU204
Trichiurus Trichiurus brevis BBG416033 OTU247
Trichiurus Trichiurus brevis BBG416039 OTU247
Trichiurus Trichiurus brevis KY372254 OTU247
Trichiurus Trichiurus brevis KY372255 OTU247
Trichiurus Trichiurus japonicus KY372288 OTU36
Trichiurus Trichiurus japonicus KY372284 OTU36
Trichiurus Trichiurus japonicus KY372286 OTU36
Trichiurus Trichiurus japonicus GOU100411 OTU36
Lepturacanthus Lepturacanthus savala KY371661 OTU110
Lepturacanthus Lepturacanthus savala KY371663 OTU110
Lepturacanthus Lepturacanthus savala GOU101098 OTU110
Lepturacanthus Lepturacanthus savala KY371668 OTU110
Lepturacanthus Lepturacanthus savala KY371667 OTU110
Lepturacanthus Lepturacanthus savala KY371665 OTU110
Lepturacanthus Lepturacanthus savala KY371669 OTU110
Lepturacanthus Lepturacanthus savala KY371662 OTU110
Rastrelliger Rastrelliger kanagurta KY372073 OTU52
Rastrelliger Rastrelliger kanagurta KY372080 OTU52
Rastrelliger Rastrelliger kanagurta KY372077 OTU52
Rastrelliger Rastrelliger kanagurta ZBL000428 OTU52
Rastrelliger Rastrelliger kanagurta GOU100503 OTU52
Rastrelliger Rastrelliger kanagurta KY372081 OTU52
Rastrelliger Rastrelliger kanagurta KY372076 OTU52
Rastrelliger Rastrelliger kanagurta KY372082 OTU52
Rastrelliger Rastrelliger kanagurta KY372072 OTU52

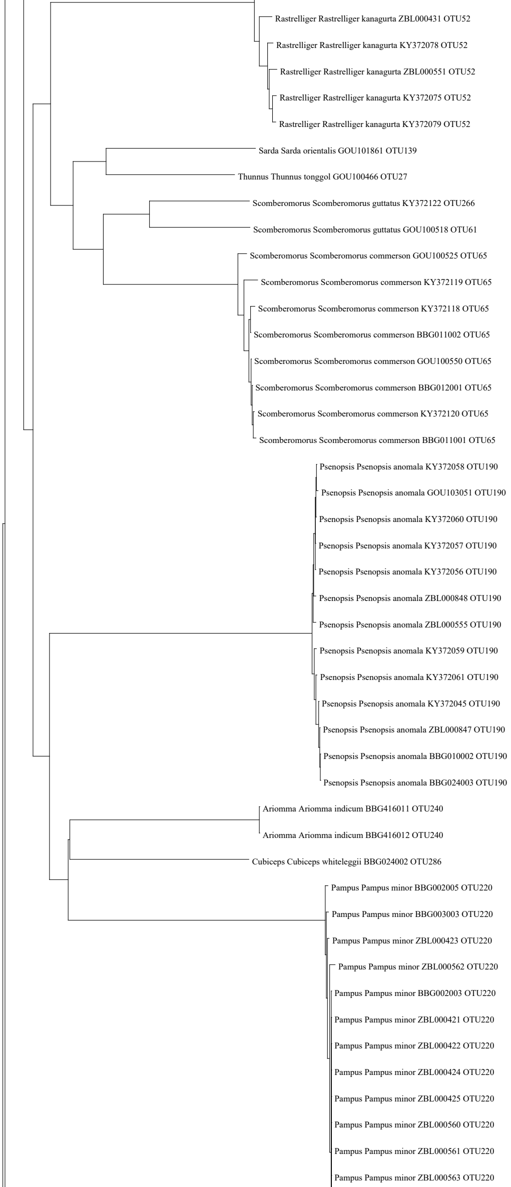

Rastrelliger Rastrelliger kanagurta ZBL000431 OTU52
Rastrelliger Rastrelliger kanagurta KY372078 OTU52
Rastrelliger Rastrelliger kanagurta ZBL000551 OTU52
Rastrelliger Rastrelliger kanagurta KY372075 OTU52
Rastrelliger Rastrelliger kanagurta KY372079 OTU52
Sarda Sarda orientalis GOU101861 OTU139
Thunnus Thunnus tonggol GOU100466 OTU27
Scomberomorus Scomberomorus guttatus KY372122 OTU266
Scomberomorus Scomberomorus guttatus GOU100518 OTU61
Scomberomorus Scomberomorus commerson GOU100525 OTU65
Scomberomorus Scomberomorus commerson KY372119 OTU65
Scomberomorus Scomberomorus commerson KY372118 OTU65
Scomberomorus Scomberomorus commerson BBG011002 OTU65
Scomberomorus Scomberomorus commerson GOU100550 OTU65
Scomberomorus Scomberomorus commerson BBG012001 OTU65
Scomberomorus Scomberomorus commerson KY372120 OTU65
Scomberomorus Scomberomorus commerson BBG011001 OTU65
Psenopsis Psenopsis anomala KY372058 OTU190
Psenopsis Psenopsis anomala GOU103051 OTU190
Psenopsis Psenopsis anomala KY372060 OTU190
Psenopsis Psenopsis anomala KY372057 OTU190
Psenopsis Psenopsis anomala KY372056 OTU190
Psenopsis Psenopsis anomala ZBL000848 OTU190
Psenopsis Psenopsis anomala ZBL000555 OTU190
Psenopsis Psenopsis anomala KY372059 OTU190
Psenopsis Psenopsis anomala KY372061 OTU190
Psenopsis Psenopsis anomala KY372045 OTU190
Psenopsis Psenopsis anomala ZBL000847 OTU190
Psenopsis Psenopsis anomala BBG010002 OTU190
Psenopsis Psenopsis anomala BBG024003 OTU190
Ariomma Ariomma indicum BBG416011 OTU240
Ariomma Ariomma indicum BBG416012 OTU240
Cubiceps Cubiceps whiteleggii BBG024002 OTU286
Pampus Pampus minor BBG002005 OTU220
Pampus Pampus minor BBG003003 OTU220
Pampus Pampus minor ZBL000423 OTU220
Pampus Pampus minor ZBL000562 OTU220
Pampus Pampus minor BBG002003 OTU220
Pampus Pampus minor ZBL000421 OTU220
Pampus Pampus minor ZBL000422 OTU220
Pampus Pampus minor ZBL000424 OTU220
Pampus Pampus minor ZBL000425 OTU220
Pampus Pampus minor ZBL000560 OTU220
Pampus Pampus minor ZBL000561 OTU220
Pampus Pampus minor ZBL000563 OTU220

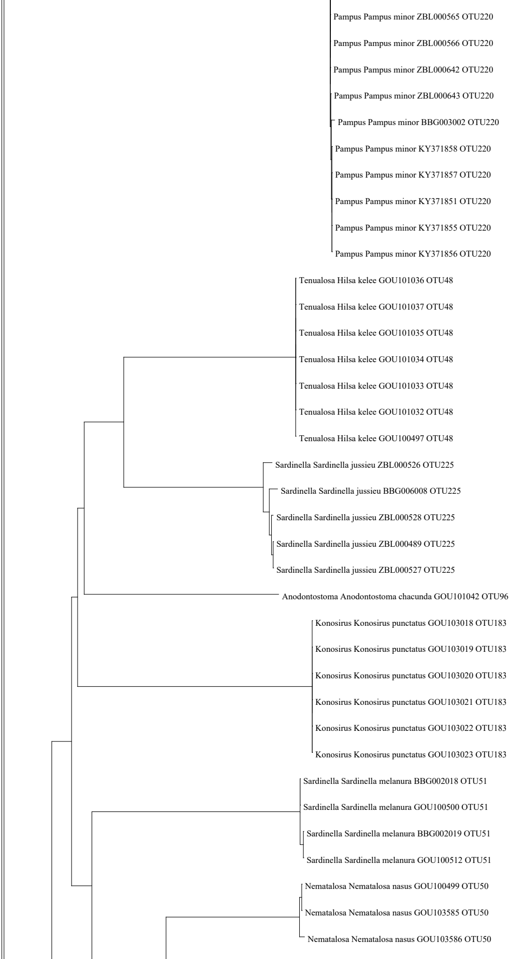
Pampus Pampus minor ZBL000565 OTU220
Pampus Pampus minor ZBL000566 OTU220
Pampus Pampus minor ZBL000642 OTU220
Pampus Pampus minor ZBL000643 OTU220
Pampus Pampus minor BBG003002 OTU220
Pampus Pampus minor KY371858 OTU220
Pampus Pampus minor KY371857 OTU220
Pampus Pampus minor KY371851 OTU220
Pampus Pampus minor KY371855 OTU220
Pampus Pampus minor KY371856 OTU220
Tenualosa Hilsa kelee GOU101036 OTU48
Tenualosa Hilsa kelee GOU101037 OTU48
Tenualosa Hilsa kelee GOU101035 OTU48
Tenualosa Hilsa kelee GOU101034 OTU48
Tenualosa Hilsa kelee GOU101033 OTU48
Tenualosa Hilsa kelee GOU101032 OTU48
Tenualosa Hilsa kelee GOU100497 OTU48
Sardinella Sardinella jussieu ZBL000526 OTU225
Sardinella Sardinella jussieu BBG006008 OTU225
Sardinella Sardinella jussieu ZBL000528 OTU225
Sardinella Sardinella jussieu ZBL000489 OTU225
Sardinella Sardinella jussieu ZBL000527 OTU225
Anodontostoma Anodontostoma chacunda GOU101042 OTU96
Konosirus Konosirus punctatus GOU103018 OTU183
Konosirus Konosirus punctatus GOU103019 OTU183
Konosirus Konosirus punctatus GOU103020 OTU183
Konosirus Konosirus punctatus GOU103021 OTU183
Konosirus Konosirus punctatus GOU103022 OTU183
Konosirus Konosirus punctatus GOU103023 OTU183
Sardinella Sardinella melanura BBG002018 OTU51
Sardinella Sardinella melanura GOU100500 OTU51
Sardinella Sardinella melanura BBG002019 OTU51
Sardinella Sardinella melanura GOU100512 OTU51
Nematalosa Nematalosa nasus GOU100499 OTU50
Nematalosa Nematalosa nasus GOU103585 OTU50
Nematalosa Nematalosa nasus GOU103586 OTU50

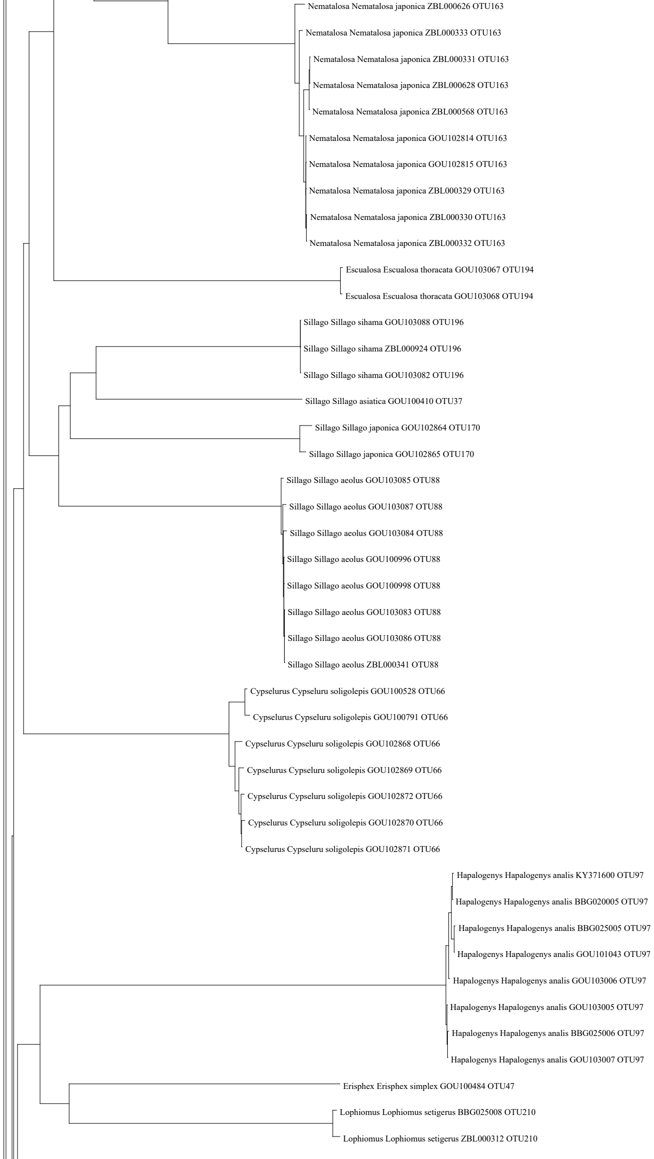

Nematalosa Nematalosa japonica ZBL000626 OTU163
Nematalosa Nematalosa japonica ZBL000333 OTU163
Nematalosa Nematalosa japonica ZBL000331 OTU163
Nematalosa Nematalosa japonica ZBL000628 OTU163
Nematalosa Nematalosa japonica ZBL000568 OTU163
Nematalosa Nematalosa japonica GOU102814 OTU163
Nematalosa Nematalosa japonica GOU102815 OTU163
Nematalosa Nematalosa japonica ZBL000329 OTU163
Nematalosa Nematalosa japonica ZBL000330 OTU163
Nematalosa Nematalosa japonica ZBL000332 OTU163
Escualosa Escualosa thoracata GOU103067 OTU194
Escualosa Escualosa thoracata GOU103068 OTU194
Sillago Sillago sihama GOU103088 OTU196
Sillago Sillago sihama ZBL000924 OTU196
Sillago Sillago sihama GOU103082 OTU196
Sillago Sillago asiatica GOU100410 OTU37
Sillago Sillago japonica GOU102864 OTU170
Sillago Sillago japonica GOU102865 OTU170
Sillago Sillago aeolus GOU103085 OTU88
Sillago Sillago aeolus GOU103087 OTU88
Sillago Sillago aeolus GOU103084 OTU88
Sillago Sillago aeolus GOU100996 OTU88
Sillago Sillago aeolus GOU100998 OTU88
Sillago Sillago aeolus GOU103083 OTU88
Sillago Sillago aeolus GOU103086 OTU88
Sillago Sillago aeolus ZBL000341 OTU88
Cypselurus Cypseluru soligolepis GOU100528 OTU66
Cypselurus Cypseluru soligolepis GOU100791 OTU66
Cypselurus Cypseluru soligolepis GOU102868 OTU66
Cypselurus Cypseluru soligolepis GOU102869 OTU66
Cypselurus Cypseluru soligolepis GOU102872 OTU66
Cypselurus Cypseluru soligolepis GOU102870 OTU66
Cypselurus Cypseluru soligolepis GOU102871 OTU66
Hapalogenys Hapalogenys analis KY371600 OTU97
Hapalogenys Hapalogenys analis BBG020005 OTU97
Hapalogenys Hapalogenys analis BBG025005 OTU97
Hapalogenys Hapalogenys analis GOU101043 OTU97
Hapalogenys Hapalogenys analis GOU103006 OTU97
Hapalogenys Hapalogenys analis GOU103005 OTU97
Hapalogenys Hapalogenys analis BBG025006 OTU97
Hapalogenys Hapalogenys analis GOU103007 OTU97
Erisphex Erisphex simplex GOU100484 OTU47
Lophiomus Lophiomus setigerus BBG025008 OTU210
Lophiomus Lophiomus setigerus ZBL000312 OTU210

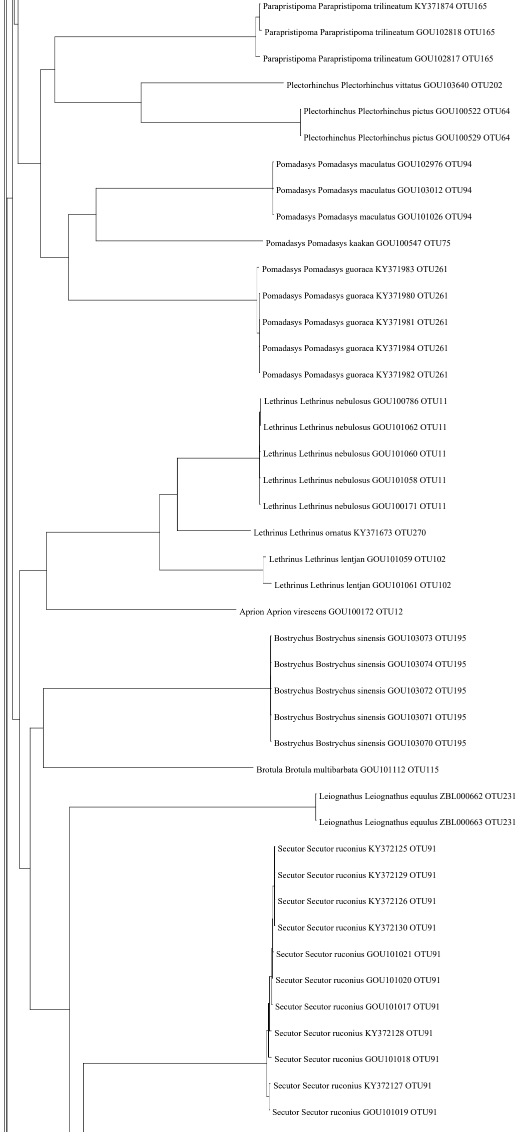
Parapristipoma Parapristipoma trilineatum KY371874 OTU165
Parapristipoma Parapristipoma trilineatum GOU102818 OTU165
Parapristipoma Parapristipoma trilineatum GOU102817 OTU165
Plectorhinchus Plectorhinchus vittatus GOU103640 OTU202
Plectorhinchus Plectorhinchus pictus GOU100522 OTU64
Plectorhinchus Plectorhinchus pictus GOU100529 OTU64
Pomadasys Pomadasys maculatus GOU102976 OTU94
Pomadasys Pomadasys maculatus GOU103012 OTU94
Pomadasys Pomadasys maculatus GOU101026 OTU94
Pomadasys Pomadasys kaakan GOU100547 OTU75
Pomadasys Pomadasys guoraca KY371983 OTU261
Pomadasys Pomadasys guoraca KY371980 OTU261
Pomadasys Pomadasys guoraca KY371981 OTU261
Pomadasys Pomadasys guoraca KY371984 OTU261
Pomadasys Pomadasys guoraca KY371982 OTU261
Lethrinus Lethrinus nebulosus GOU100786 OTU11
Lethrinus Lethrinus nebulosus GOU101062 OTU11
Lethrinus Lethrinus nebulosus GOU101060 OTU11
Lethrinus Lethrinus nebulosus GOU101058 OTU11
Lethrinus Lethrinus nebulosus GOU100171 OTU11
Lethrinus Lethrinus ornatus KY371673 OTU270
Lethrinus Lethrinus lentjan GOU101059 OTU102
Lethrinus Lethrinus lentjan GOU101061 OTU102
Aprion Aprion virescens GOU100172 OTU12
Bostrychus Bostrychus sinensis GOU103073 OTU195
Bostrychus Bostrychus sinensis GOU103074 OTU195
Bostrychus Bostrychus sinensis GOU103072 OTU195
Bostrychus Bostrychus sinensis GOU103071 OTU195
Bostrychus Bostrychus sinensis GOU103070 OTU195
Brotula Brotula multibarbata GOU101112 OTU115
Leiognathus Leiognathus equulus ZBL000662 OTU231
Leiognathus Leiognathus equulus ZBL000663 OTU231
Secutor Secutor ruconius KY372125 OTU91
Secutor Secutor ruconius KY372129 OTU91
Secutor Secutor ruconius KY372126 OTU91
Secutor Secutor ruconius KY372130 OTU91
Secutor Secutor ruconius GOU101021 OTU91
Secutor Secutor ruconius GOU101020 OTU91
Secutor Secutor ruconius GOU101017 OTU91
Secutor Secutor ruconius KY372128 OTU91
Secutor Secutor ruconius GOU101018 OTU91
Secutor Secutor ruconius KY372127 OTU91
Secutor Secutor ruconius GOU101019 OTU91

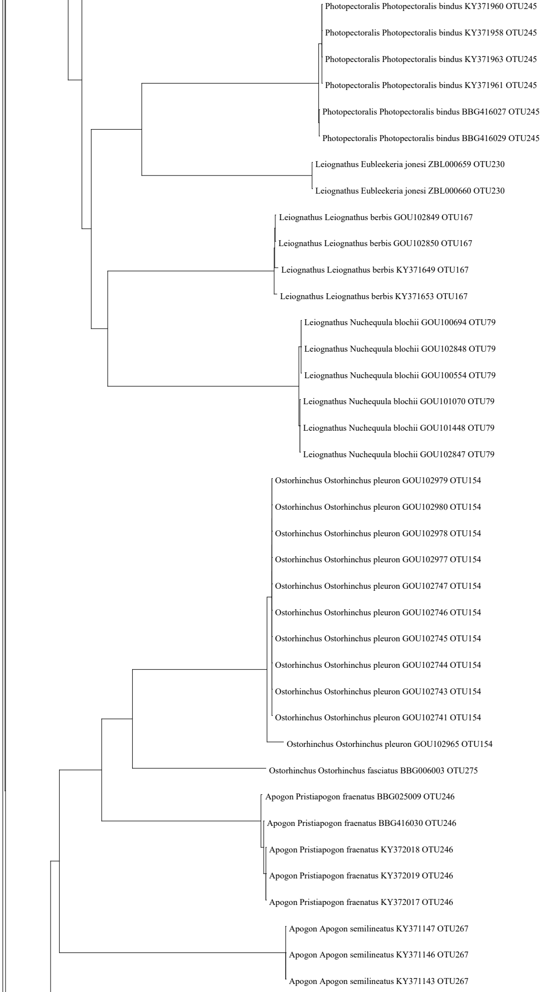
Photopectoralis Photopectoralis bindus KY371960 OTU245
Photopectoralis Photopectoralis bindus KY371958 OTU245
Photopectoralis Photopectoralis bindus KY371963 OTU245
Photopectoralis Photopectoralis bindus KY371961 OTU245
Photopectoralis Photopectoralis bindus BBG416027 OTU245
Photopectoralis Photopectoralis bindus BBG416029 OTU245
Leiognathus Eubleekeria jonesi ZBL000659 OTU230
Leiognathus Eubleekeria jonesi ZBL000660 OTU230
Leiognathus Leiognathus berbis GOU102849 OTU167
Leiognathus Leiognathus berbis GOU102850 OTU167
Leiognathus Leiognathus berbis KY371649 OTU167
Leiognathus Leiognathus berbis KY371653 OTU167
Leiognathus Nuchequula blochii GOU100694 OTU79
Leiognathus Nuchequula blochii GOU102848 OTU79
Leiognathus Nuchequula blochii GOU100554 OTU79
Leiognathus Nuchequula blochii GOU101070 OTU79
Leiognathus Nuchequula blochii GOU101448 OTU79
Leiognathus Nuchequula blochii GOU102847 OTU79
Ostorhinchus Ostorhinchus pleuron GOU102979 OTU154
Ostorhinchus Ostorhinchus pleuron GOU102980 OTU154
Ostorhinchus Ostorhinchus pleuron GOU102978 OTU154
Ostorhinchus Ostorhinchus pleuron GOU102977 OTU154
Ostorhinchus Ostorhinchus pleuron GOU102747 OTU154
Ostorhinchus Ostorhinchus pleuron GOU102746 OTU154
Ostorhinchus Ostorhinchus pleuron GOU102745 OTU154
Ostorhinchus Ostorhinchus pleuron GOU102744 OTU154
Ostorhinchus Ostorhinchus pleuron GOU102743 OTU154
Ostorhinchus Ostorhinchus pleuron GOU102741 OTU154
Ostorhinchus Ostorhinchus pleuron GOU102965 OTU154
Ostorhinchus Ostorhinchus fasciatus BBG006003 OTU275
Apogon Pristiapogon fraenatus BBG025009 OTU246
Apogon Pristiapogon fraenatus BBG416030 OTU246
Apogon Pristiapogon fraenatus KY372018 OTU246
Apogon Pristiapogon fraenatus KY372019 OTU246
Apogon Pristiapogon fraenatus KY372017 OTU246
Apogon Apogon semilineatus KY371147 OTU267
Apogon Apogon semilineatus KY371146 OTU267
Apogon Apogon semilineatus KY371143 OTU267

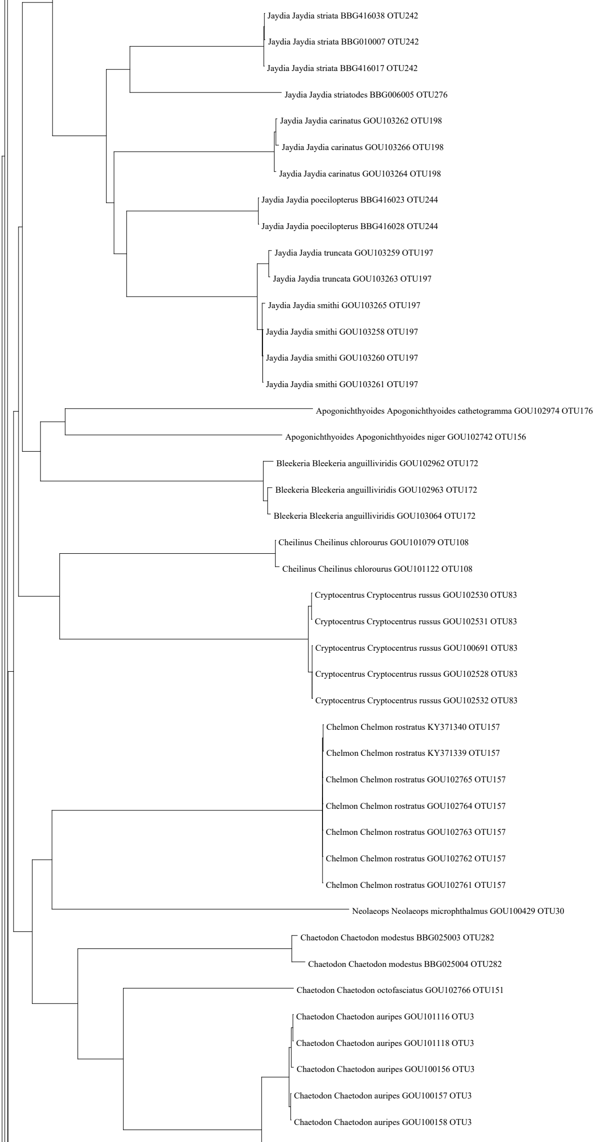
Jaydia Jaydia striata BBG416038 OTU242
Jaydia Jaydia striata BBG010007 OTU242
Jaydia Jaydia striata BBG416017 OTU242
Jaydia Jaydia striatodes BBG006005 OTU276
Jaydia Jaydia carinatus GOU103262 OTU198
Jaydia Jaydia carinatus GOU103266 OTU198
Jaydia Jaydia carinatus GOU103264 OTU198
Jaydia Jaydia poecilopterus BBG416023 OTU244
Jaydia Jaydia poecilopterus BBG416028 OTU244
Jaydia Jaydia truncata GOU103259 OTU197
Jaydia Jaydia truncata GOU103263 OTU197
Jaydia Jaydia smithi GOU103265 OTU197
Jaydia Jaydia smithi GOU103258 OTU197
Jaydia Jaydia smithi GOU103260 OTU197
Jaydia Jaydia smithi GOU103261 OTU197
Apogonichthyoides Apogonichthyoides cathetogramma GOU102974 OTU176
Apogonichthyoides Apogonichthyoides niger GOU102742 OTU156
Bleekeria Bleekeria anguilliviridis GOU102962 OTU172
Bleekeria Bleekeria anguilliviridis GOU102963 OTU172
Bleekeria Bleekeria anguilliviridis GOU103064 OTU172
Cheilinus Cheilinus chlorourus GOU101079 OTU108
Cheilinus Cheilinus chlorourus GOU101122 OTU108
Cryptocentrus Cryptocentrus russus GOU102530 OTU83
Cryptocentrus Cryptocentrus russus GOU102531 OTU83
Cryptocentrus Cryptocentrus russus GOU100691 OTU83
Cryptocentrus Cryptocentrus russus GOU102528 OTU83
Cryptocentrus Cryptocentrus russus GOU102532 OTU83
Chelmon Chelmon rostratus KY371340 OTU157
Chelmon Chelmon rostratus KY371339 OTU157
Chelmon Chelmon rostratus GOU102765 OTU157
Chelmon Chelmon rostratus GOU102764 OTU157
Chelmon Chelmon rostratus GOU102763 OTU157
Chelmon Chelmon rostratus GOU102762 OTU157
Chelmon Chelmon rostratus GOU102761 OTU157
Neolaeops Neolaeops microphthalmus GOU100429 OTU30
Chaetodon Chaetodon modestus BBG025003 OTU282
Chaetodon Chaetodon modestus BBG025004 OTU282
Chaetodon Chaetodon octofasciatus GOU102766 OTU151
Chaetodon Chaetodon auripes GOU101116 OTU3
Chaetodon Chaetodon auripes GOU101118 OTU3
Chaetodon Chaetodon auripes GOU100156 OTU3
Chaetodon Chaetodon auripes GOU100157 OTU3
Chaetodon Chaetodon auripes GOU100158 OTU3

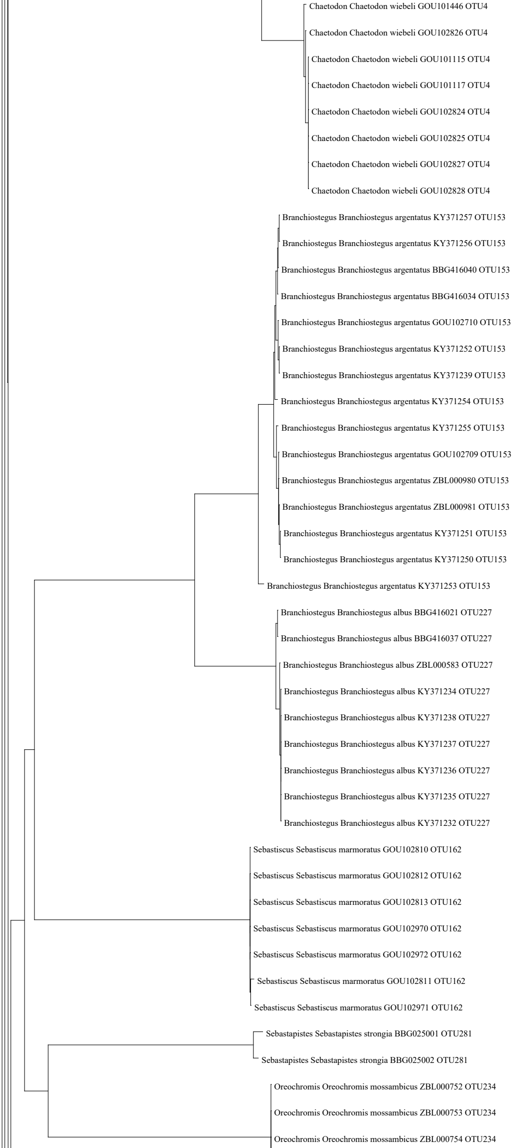
Chaetodon Chaetodon wiebeli GOU101446 OTU4
Chaetodon Chaetodon wiebeli GOU102826 OTU4
Chaetodon Chaetodon wiebeli GOU101115 OTU4
Chaetodon Chaetodon wiebeli GOU101117 OTU4
Chaetodon Chaetodon wiebeli GOU102824 OTU4
Chaetodon Chaetodon wiebeli GOU102825 OTU4
Chaetodon Chaetodon wiebeli GOU102827 OTU4
Chaetodon Chaetodon wiebeli GOU102828 OTU4
Branchiostegus Branchiostegus argentatus KY371257 OTU153
Branchiostegus Branchiostegus argentatus KY371256 OTU153
Branchiostegus Branchiostegus argentatus BBG416040 OTU153
Branchiostegus Branchiostegus argentatus BBG416034 OTU153
Branchiostegus Branchiostegus argentatus GOU102710 OTU153
Branchiostegus Branchiostegus argentatus KY371252 OTU153
Branchiostegus Branchiostegus argentatus KY371239 OTU153
Branchiostegus Branchiostegus argentatus KY371254 OTU153
Branchiostegus Branchiostegus argentatus KY371255 OTU153
Branchiostegus Branchiostegus argentatus GOU102709 OTU153
Branchiostegus Branchiostegus argentatus ZBL000980 OTU153
Branchiostegus Branchiostegus argentatus ZBL000981 OTU153
Branchiostegus Branchiostegus argentatus KY371251 OTU153
Branchiostegus Branchiostegus argentatus KY371250 OTU153
Branchiostegus Branchiostegus argentatus KY371253 OTU153
Branchiostegus Branchiostegus albus BBG416021 OTU227
Branchiostegus Branchiostegus albus BBG416037 OTU227
Branchiostegus Branchiostegus albus ZBL000583 OTU227
Branchiostegus Branchiostegus albus KY371234 OTU227
Branchiostegus Branchiostegus albus KY371238 OTU227
Branchiostegus Branchiostegus albus KY371237 OTU227
Branchiostegus Branchiostegus albus KY371236 OTU227
Branchiostegus Branchiostegus albus KY371235 OTU227
Branchiostegus Branchiostegus albus KY371232 OTU227
Sebastiscus Sebastiscus marmoratus GOU102810 OTU162
Sebastiscus Sebastiscus marmoratus GOU102812 OTU162
Sebastiscus Sebastiscus marmoratus GOU102813 OTU162
Sebastiscus Sebastiscus marmoratus GOU102970 OTU162
Sebastiscus Sebastiscus marmoratus GOU102972 OTU162
Sebastiscus Sebastiscus marmoratus GOU102811 OTU162
Sebastiscus Sebastiscus marmoratus GOU102971 OTU162
Sebastapistes Sebastapistes strongia BBG025001 OTU281
Sebastapistes Sebastapistes strongia BBG025002 OTU281
Oreochromis Oreochromis mossambicus ZBL000752 OTU234
Oreochromis Oreochromis mossambicus ZBL000753 OTU234
Oreochromis Oreochromis mossambicus ZBL000754 OTU234

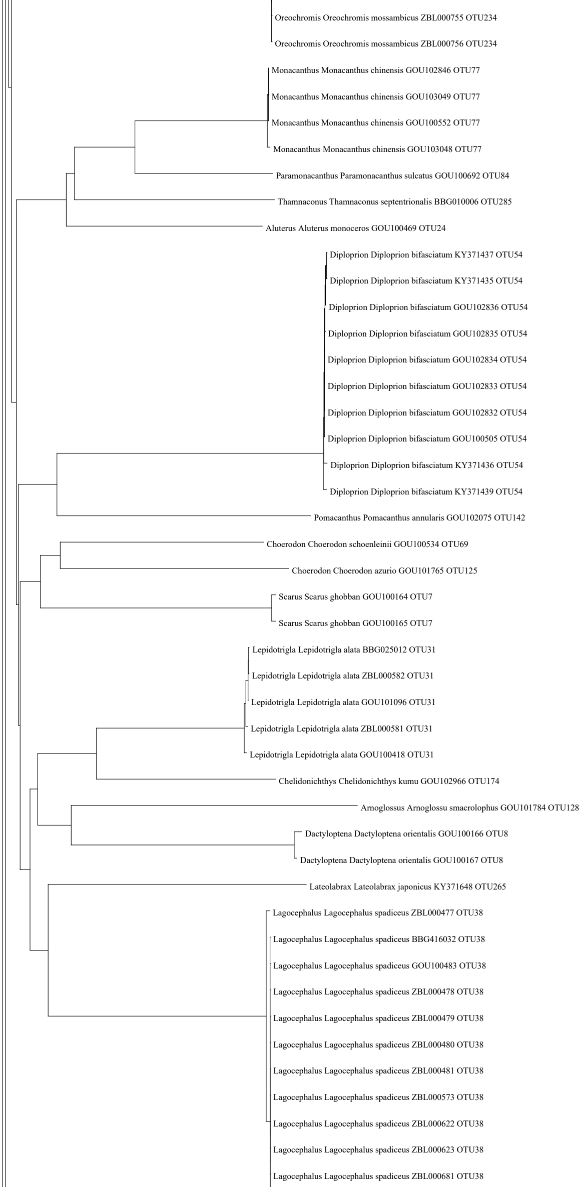
Oreochromis Oreochromis mossambicus ZBL000755 OTU234
Oreochromis Oreochromis mossambicus ZBL000756 OTU234
Monacanthus Monacanthus chinensis GOU102846 OTU77
Monacanthus Monacanthus chinensis GOU103049 OTU77
Monacanthus Monacanthus chinensis GOU100552 OTU77
Monacanthus Monacanthus chinensis GOU103048 OTU77
Paramonacanthus Paramonacanthus sulcatus GOU100692 OTU84
Thamnaconus Thamnaconus septentrionalis BBG010006 OTU285
Aluterus Aluterus monoceros GOU100469 OTU24
Diploprion Diploprion bifasciatum KY371437 OTU54
Diploprion Diploprion bifasciatum KY371435 OTU54
Diploprion Diploprion bifasciatum GOU102836 OTU54
Diploprion Diploprion bifasciatum GOU102835 OTU54
Diploprion Diploprion bifasciatum GOU102834 OTU54
Diploprion Diploprion bifasciatum GOU102833 OTU54
Diploprion Diploprion bifasciatum GOU102832 OTU54
Diploprion Diploprion bifasciatum GOU100505 OTU54
Diploprion Diploprion bifasciatum KY371436 OTU54
Diploprion Diploprion bifasciatum KY371439 OTU54
Pomacanthus Pomacanthus annularis GOU102075 OTU142
Choerodon Choerodon schoenleinii GOU100534 OTU69
Choerodon Choerodon azurio GOU101765 OTU125
Scarus Scarus ghobban GOU100164 OTU7
Scarus Scarus ghobban GOU100165 OTU7
Lepidotrigla Lepidotrigla alata BBG025012 OTU31
Lepidotrigla Lepidotrigla alata ZBL000582 OTU31
Lepidotrigla Lepidotrigla alata GOU101096 OTU31
Lepidotrigla Lepidotrigla alata ZBL000581 OTU31
Lepidotrigla Lepidotrigla alata GOU100418 OTU31
Chelidonichthys Chelidonichthys kumu GOU102966 OTU174
Arnoglossus Arnoglossu smacrolophus GOU101784 OTU128
Dactyloptena Dactyloptena orientalis GOU100166 OTU8
Dactyloptena Dactyloptena orientalis GOU100167 OTU8
Lateolabrax Lateolabrax japonicus KY371648 OTU265
Lagocephalus Lagocephalus spadiceus ZBL000477 OTU38
Lagocephalus Lagocephalus spadiceus BBG416032 OTU38
Lagocephalus Lagocephalus spadiceus GOU100483 OTU38
Lagocephalus Lagocephalus spadiceus ZBL000478 OTU38
Lagocephalus Lagocephalus spadiceus ZBL000479 OTU38
Lagocephalus Lagocephalus spadiceus ZBL000480 OTU38
Lagocephalus Lagocephalus spadiceus ZBL000481 OTU38
Lagocephalus Lagocephalus spadiceus ZBL000573 OTU38
Lagocephalus Lagocephalus spadiceus ZBL000622 OTU38
Lagocephalus Lagocephalus spadiceus ZBL000623 OTU38
Lagocephalus Lagocephalus spadiceus ZBL000681 OTU38

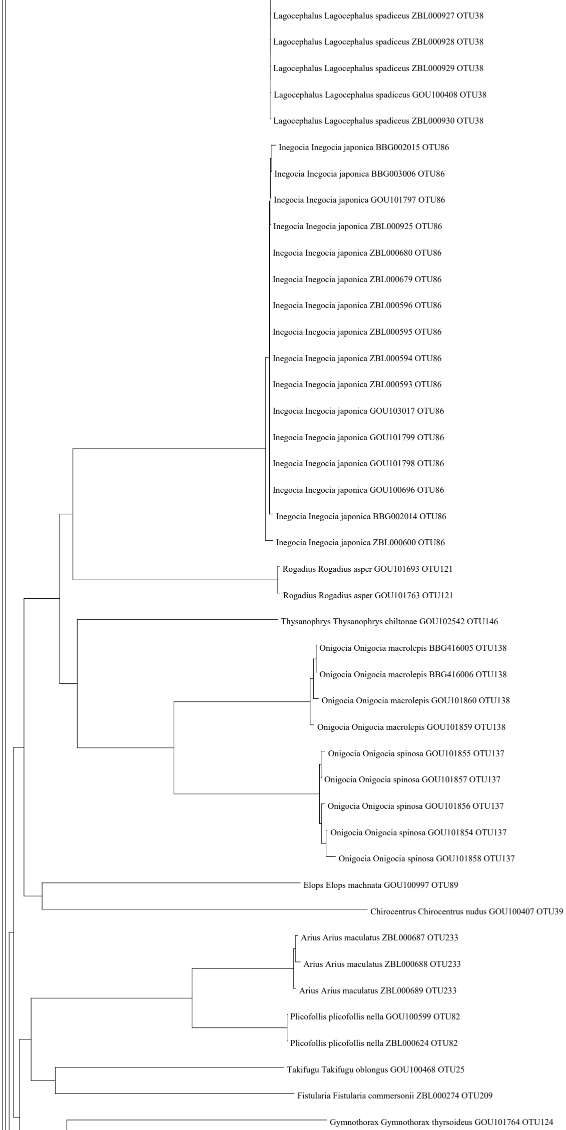
Lagocephalus Lagocephalus spadiceus ZBL000927 OTU38
Lagocephalus Lagocephalus spadiceus ZBL000928 OTU38
Lagocephalus Lagocephalus spadiceus ZBL000929 OTU38
Lagocephalus Lagocephalus spadiceus GOU100408 OTU38
Lagocephalus Lagocephalus spadiceus ZBL000930 OTU38
Inegocia Inegocia japonica BBG002015 OTU86
Inegocia Inegocia japonica BBG003006 OTU86
Inegocia Inegocia japonica GOU101797 OTU86
Inegocia Inegocia japonica ZBL000925 OTU86
Inegocia Inegocia japonica ZBL000680 OTU86
Inegocia Inegocia japonica ZBL000679 OTU86
Inegocia Inegocia japonica ZBL000596 OTU86
Inegocia Inegocia japonica ZBL000595 OTU86
Inegocia Inegocia japonica ZBL000594 OTU86
Inegocia Inegocia japonica ZBL000593 OTU86
Inegocia Inegocia japonica GOU103017 OTU86
Inegocia Inegocia japonica GOU101799 OTU86
Inegocia Inegocia japonica GOU101798 OTU86
Inegocia Inegocia japonica GOU100696 OTU86
Inegocia Inegocia japonica BBG002014 OTU86
Inegocia Inegocia japonica ZBL000600 OTU86
Rogadius Rogadius asper GOU101693 OTU121
Rogadius Rogadius asper GOU101763 OTU121
Thysanophrys Thysanophrys chiltonae GOU102542 OTU146
Onigocia Onigocia macrolepis BBG416005 OTU138
Onigocia Onigocia macrolepis BBG416006 OTU138
Onigocia Onigocia macrolepis GOU101860 OTU138
Onigocia Onigocia macrolepis GOU101859 OTU138
Onigocia Onigocia spinosa GOU101855 OTU137
Onigocia Onigocia spinosa GOU101857 OTU137
Onigocia Onigocia spinosa GOU101856 OTU137
Onigocia Onigocia spinosa GOU101854 OTU137
Onigocia Onigocia spinosa GOU101858 OTU137
Elops Elops machnata GOU100997 OTU89
Chirocentrus Chirocentrus nudus GOU100407 OTU39
Arius Arius maculatus ZBL000687 OTU233
Arius Arius maculatus ZBL000688 OTU233
Arius Arius maculatus ZBL000689 OTU233
Plicofollis plicofollis nella GOU100599 OTU82
Plicofollis plicofollis nella ZBL000624 OTU82
Takifugu Takifugu oblongus GOU100468 OTU25
Fistularia Fistularia commersonii ZBL000274 OTU209
Gymnothorax Gymnothorax thyrsoideus GOU101764 OTU124

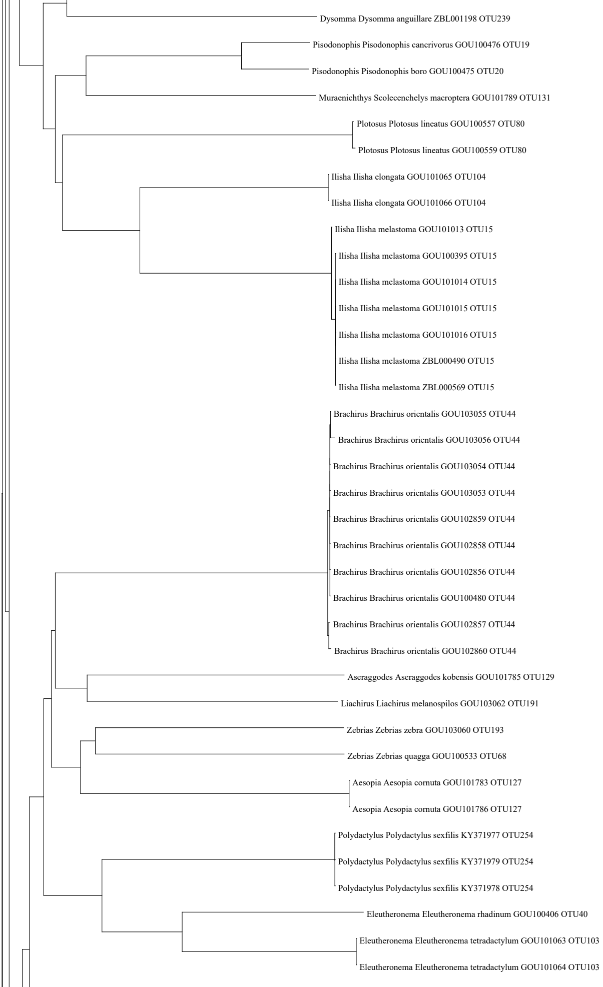

Dysomma Dysomma anguillare ZBL001198 OTU239
Pisodonophis Pisodonophis cancrivorus GOU100476 OTU19
Pisodonophis Pisodonophis boro GOU100475 OTU20
Muraenichthys Scolecenchelys macroptera GOU101789 OTU131
Plotosus Plotosus lineatus GOU100557 OTU80
Plotosus Plotosus lineatus GOU100559 OTU80
Ilisha Ilisha elongata GOU101065 OTU104
Ilisha Ilisha elongata GOU101066 OTU104
Ilisha Ilisha melastoma GOU101013 OTU15
Ilisha Ilisha melastoma GOU100395 OTU15
Ilisha Ilisha melastoma GOU101014 OTU15
Ilisha Ilisha melastoma GOU101015 OTU15
Ilisha Ilisha melastoma GOU101016 OTU15
Ilisha Ilisha melastoma ZBL000490 OTU15
Ilisha Ilisha melastoma ZBL000569 OTU15
Brachirus Brachirus orientalis GOU103055 OTU44
Brachirus Brachirus orientalis GOU103056 OTU44
Brachirus Brachirus orientalis GOU103054 OTU44
Brachirus Brachirus orientalis GOU103053 OTU44
Brachirus Brachirus orientalis GOU102859 OTU44
Brachirus Brachirus orientalis GOU102858 OTU44
Brachirus Brachirus orientalis GOU102856 OTU44
Brachirus Brachirus orientalis GOU100480 OTU44
Brachirus Brachirus orientalis GOU102857 OTU44
Brachirus Brachirus orientalis GOU102860 OTU44
Aseraggodes Aseraggodes kobensis GOU101785 OTU129
Liachirus Liachirus melanospilos GOU103062 OTU191
Zebrias Zebrias zebra GOU103060 OTU193
Zebrias Zebrias quagga GOU100533 OTU68
Aesopia Aesopia cornuta GOU101783 OTU127
Aesopia Aesopia cornuta GOU101786 OTU127
Polydactylus Polydactylus sexfilis KY371977 OTU254
Polydactylus Polydactylus sexfilis KY371979 OTU254
Polydactylus Polydactylus sexfilis KY371978 OTU254
Eleutheronema Eleutheronema rhadinum GOU100406 OTU40
Eleutheronema Eleutheronema tetradactylum GOU101063 OTU103
Eleutheronema Eleutheronema tetradactylum GOU101064 OTU103

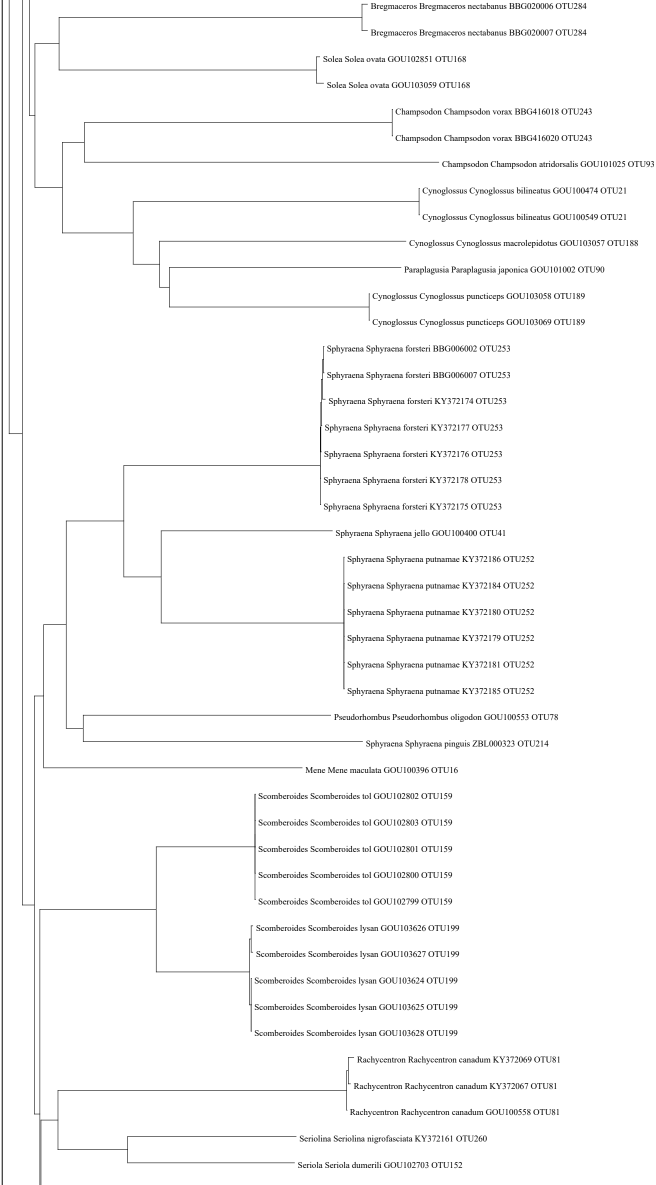
Bregmaceros Bregmaceros nectabanus BBG020006 OTU284
Bregmaceros Bregmaceros nectabanus BBG020007 OTU284
Solea Solea ovata GOU102851 OTU168
Solea Solea ovata GOU103059 OTU168
Champsodon Champsodon vorax BBG416018 OTU243
Champsodon Champsodon vorax BBG416020 OTU243
Champsodon Champsodon atridorsalis GOU101025 OTU93
Cynoglossus Cynoglossus bilineatus GOU100474 OTU21
Cynoglossus Cynoglossus bilineatus GOU100549 OTU21
Cynoglossus Cynoglossus macrolepidotus GOU103057 OTU188
Paraplagusia Paraplagusia japonica GOU101002 OTU90
Cynoglossus Cynoglossus puncticeps GOU103058 OTU189
Cynoglossus Cynoglossus puncticeps GOU103069 OTU189
Sphyraena Sphyraena forsteri BBG006002 OTU253
Sphyraena Sphyraena forsteri BBG006007 OTU253
Sphyraena Sphyraena forsteri KY372174 OTU253
Sphyraena Sphyraena forsteri KY372177 OTU253
Sphyraena Sphyraena forsteri KY372176 OTU253
Sphyraena Sphyraena forsteri KY372178 OTU253
Sphyraena Sphyraena forsteri KY372175 OTU253
Sphyraena Sphyraena jello GOU100400 OTU41
Sphyraena Sphyraena putnamae KY372186 OTU252
Sphyraena Sphyraena putnamae KY372184 OTU252
Sphyraena Sphyraena putnamae KY372180 OTU252
Sphyraena Sphyraena putnamae KY372179 OTU252
Sphyraena Sphyraena putnamae KY372181 OTU252
Sphyraena Sphyraena putnamae KY372185 OTU252
Pseudorhombus Pseudorhombus oligodon GOU100553 OTU78
Sphyraena Sphyraena pinguis ZBL000323 OTU214
Mene Mene maculata GOU100396 OTU16
Scomberoides Scomberoides tol GOU102802 OTU159
Scomberoides Scomberoides tol GOU102803 OTU159
Scomberoides Scomberoides tol GOU102801 OTU159
Scomberoides Scomberoides tol GOU102800 OTU159
Scomberoides Scomberoides tol GOU102799 OTU159
Scomberoides Scomberoides lysan GOU103626 OTU199
Scomberoides Scomberoides lysan GOU103627 OTU199
Scomberoides Scomberoides lysan GOU103624 OTU199
Scomberoides Scomberoides lysan GOU103625 OTU199
Scomberoides Scomberoides lysan GOU103628 OTU199
Rachycentron Rachycentron canadum KY372069 OTU81
Rachycentron Rachycentron canadum KY372067 OTU81
Rachycentron Rachycentron canadum GOU100558 OTU81
Seriolina Seriolina nigrofasciata KY372161 OTU260
Seriola Seriola dumerili GOU102703 OTU152

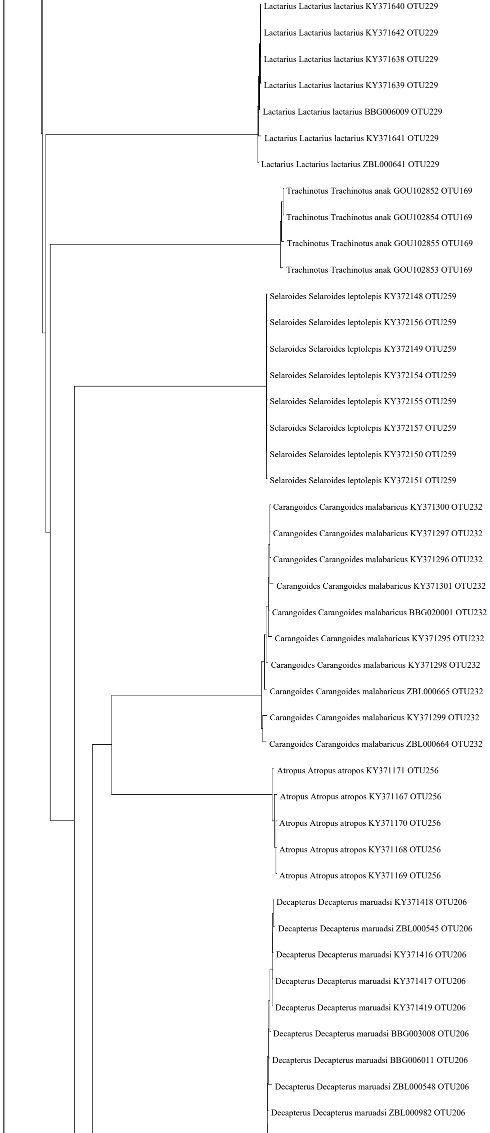
Lactarius Lactarius lactarius KY371640 OTU229
Lactarius Lactarius lactarius KY371642 OTU229
Lactarius Lactarius lactarius KY371638 OTU229
Lactarius Lactarius lactarius KY371639 OTU229
Lactarius Lactarius lactarius BBG006009 OTU229
Lactarius Lactarius lactarius KY371641 OTU229
Lactarius Lactarius lactarius ZBL000641 OTU229
Trachinotus Trachinotus anak GOU102852 OTU169
Trachinotus Trachinotus anak GOU102854 OTU169
Trachinotus Trachinotus anak GOU102855 OTU169
Trachinotus Trachinotus anak GOU102853 OTU169
Selaroides Selaroides leptolepis KY372148 OTU259
Selaroides Selaroides leptolepis KY372156 OTU259
Selaroides Selaroides leptolepis KY372149 OTU259
Selaroides Selaroides leptolepis KY372154 OTU259
Selaroides Selaroides leptolepis KY372155 OTU259
Selaroides Selaroides leptolepis KY372157 OTU259
Selaroides Selaroides leptolepis KY372150 OTU259
Selaroides Selaroides leptolepis KY372151 OTU259
Carangoides Carangoides malabaricus KY371300 OTU232
Carangoides Carangoides malabaricus KY371297 OTU232
Carangoides Carangoides malabaricus KY371296 OTU232
Carangoides Carangoides malabaricus KY371301 OTU232
Carangoides Carangoides malabaricus BBG020001 OTU232
Carangoides Carangoides malabaricus KY371295 OTU232
Carangoides Carangoides malabaricus KY371298 OTU232
Carangoides Carangoides malabaricus ZBL000665 OTU232
Carangoides Carangoides malabaricus KY371299 OTU232
Carangoides Carangoides malabaricus ZBL000664 OTU232
Atropus Atropus atropos KY371171 OTU256
Atropus Atropus atropos KY371167 OTU256
Atropus Atropus atropos KY371170 OTU256
Atropus Atropus atropos KY371168 OTU256
Atropus Atropus atropos KY371169 OTU256
Decapterus Decapterus maruadsi KY371418 OTU206
Decapterus Decapterus maruadsi ZBL000545 OTU206
Decapterus Decapterus maruadsi KY371416 OTU206
Decapterus Decapterus maruadsi KY371417 OTU206
Decapterus Decapterus maruadsi KY371419 OTU206
Decapterus Decapterus maruadsi BBG003008 OTU206
Decapterus Decapterus maruadsi BBG006011 OTU206
Decapterus Decapterus maruadsi ZBL000548 OTU206
Decapterus Decapterus maruadsi ZBL000982 OTU206

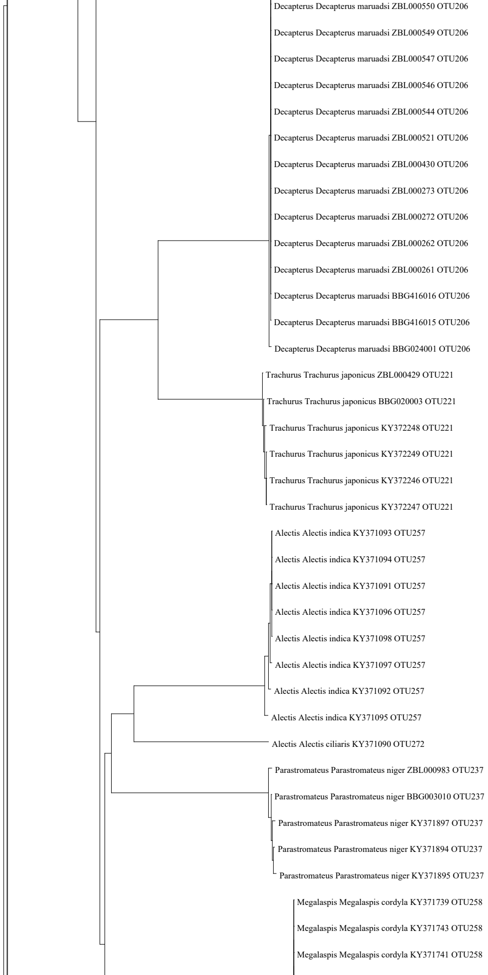
Decapterus Decapterus maruadsi ZBL000550 OTU206
Decapterus Decapterus maruadsi ZBL000549 OTU206
Decapterus Decapterus maruadsi ZBL000547 OTU206
Decapterus Decapterus maruadsi ZBL000546 OTU206
Decapterus Decapterus maruadsi ZBL000544 OTU206
Decapterus Decapterus maruadsi ZBL000521 OTU206
Decapterus Decapterus maruadsi ZBL000430 OTU206
Decapterus Decapterus maruadsi ZBL000273 OTU206
Decapterus Decapterus maruadsi ZBL000272 OTU206
Decapterus Decapterus maruadsi ZBL000262 OTU206
Decapterus Decapterus maruadsi ZBL000261 OTU206
Decapterus Decapterus maruadsi BBG416016 OTU206
Decapterus Decapterus maruadsi BBG416015 OTU206
Decapterus Decapterus maruadsi BBG024001 OTU206
Trachurus Trachurus japonicus ZBL000429 OTU221
Trachurus Trachurus japonicus BBG020003 OTU221
Trachurus Trachurus japonicus KY372248 OTU221
Trachurus Trachurus japonicus KY372249 OTU221
Trachurus Trachurus japonicus KY372246 OTU221
Trachurus Trachurus japonicus KY372247 OTU221
Alectis Alectis indica KY371093 OTU257
Alectis Alectis indica KY371094 OTU257
Alectis Alectis indica KY371091 OTU257
Alectis Alectis indica KY371096 OTU257
Alectis Alectis indica KY371098 OTU257
Alectis Alectis indica KY371097 OTU257
Alectis Alectis indica KY371092 OTU257
Alectis Alectis indica KY371095 OTU257
Alectis Alectis ciliaris KY371090 OTU272
Parastromateus Parastromateus niger ZBL000983 OTU237
Parastromateus Parastromateus niger BBG003010 OTU237
Parastromateus Parastromateus niger KY371897 OTU237
Parastromateus Parastromateus niger KY371894 OTU237
Parastromateus Parastromateus niger KY371895 OTU237
Megalaspis Megalaspis cordyla KY371739 OTU258
Megalaspis Megalaspis cordyla KY371743 OTU258
Megalaspis Megalaspis cordyla KY371741 OTU258

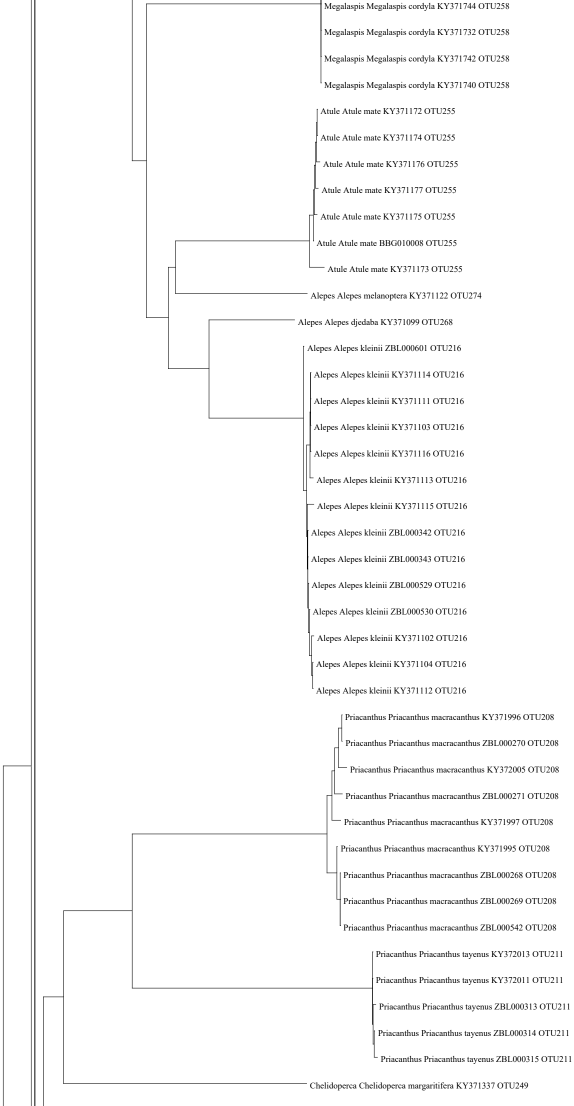
Megalaspis Megalaspis cordyla KY371744 OTU258
Megalaspis Megalaspis cordyla KY371732 OTU258
Megalaspis Megalaspis cordyla KY371742 OTU258
Megalaspis Megalaspis cordyla KY371740 OTU258
Atule Atule mate KY371172 OTU255
Atule Atule mate KY371174 OTU255
Atule Atule mate KY371176 OTU255
Atule Atule mate KY371177 OTU255
Atule Atule mate KY371175 OTU255
Atule Atule mate BBG010008 OTU255
Atule Atule mate KY371173 OTU255
Alepes Alepes melanoptera KY371122 OTU274
Alepes Alepes djedaba KY371099 OTU268
Alepes Alepes kleinii ZBL000601 OTU216
Alepes Alepes kleinii KY371114 OTU216
Alepes Alepes kleinii KY371111 OTU216
Alepes Alepes kleinii KY371103 OTU216
Alepes Alepes kleinii KY371116 OTU216
Alepes Alepes kleinii KY371113 OTU216
Alepes Alepes kleinii KY371115 OTU216
Alepes Alepes kleinii ZBL000342 OTU216
Alepes Alepes kleinii ZBL000343 OTU216
Alepes Alepes kleinii ZBL000529 OTU216
Alepes Alepes kleinii ZBL000530 OTU216
Alepes Alepes kleinii KY371102 OTU216
Alepes Alepes kleinii KY371104 OTU216
Alepes Alepes kleinii KY371112 OTU216
Priacanthus Priacanthus macracanthus KY371996 OTU208
Priacanthus Priacanthus macracanthus ZBL000270 OTU208
Priacanthus Priacanthus macracanthus KY372005 OTU208
Priacanthus Priacanthus macracanthus ZBL000271 OTU208
Priacanthus Priacanthus macracanthus KY371997 OTU208
Priacanthus Priacanthus macracanthus KY371995 OTU208
Priacanthus Priacanthus macracanthus ZBL000268 OTU208
Priacanthus Priacanthus macracanthus ZBL000269 OTU208
Priacanthus Priacanthus macracanthus ZBL000542 OTU208
Priacanthus Priacanthus tayenus KY372013 OTU211
Priacanthus Priacanthus tayenus KY372011 OTU211
Priacanthus Priacanthus tayenus ZBL000313 OTU211
Priacanthus Priacanthus tayenus ZBL000314 OTU211
Priacanthus Priacanthus tayenus ZBL000315 OTU211
Chelidoperca Chelidoperca margaritifera KY371337 OTU249

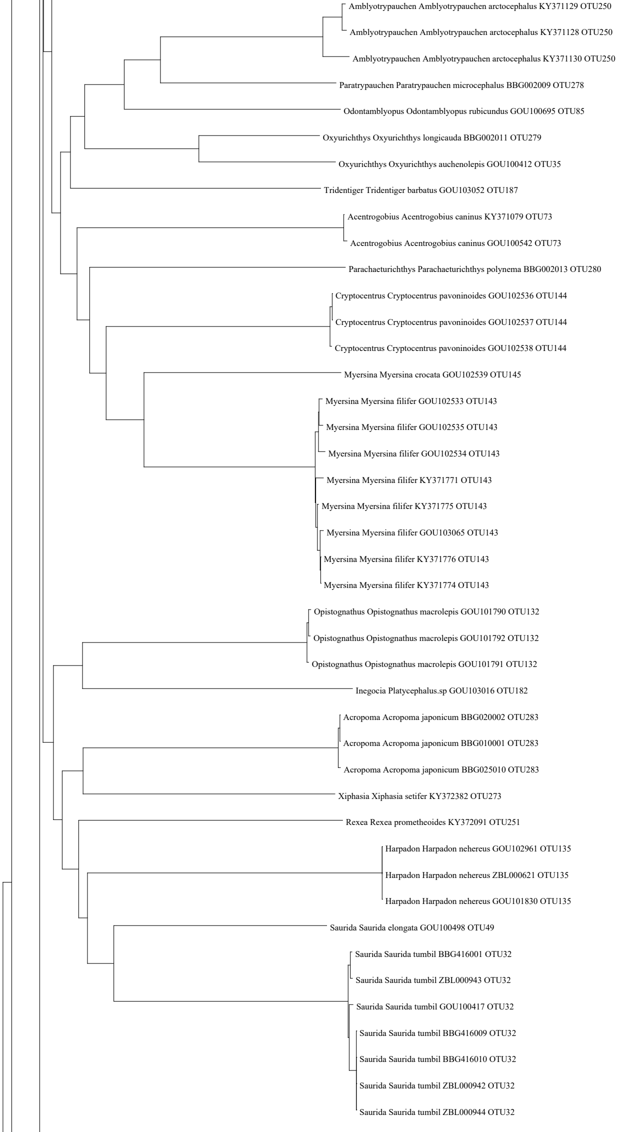
Amblyotrypauchen Amblyotrypauchen arctocephalus KY371129 OTU250
Amblyotrypauchen Amblyotrypauchen arctocephalus KY371128 OTU250
Amblyotrypauchen Amblyotrypauchen arctocephalus KY371130 OTU250
Paratrypauchen Paratrypauchen microcephalus BBG002009 OTU278
Odontamblyopus Odontamblyopus rubicundus GOU100695 OTU85
Oxyurichthys Oxyurichthys longicauda BBG002011 OTU279
Oxyurichthys Oxyurichthys auchenolepis GOU100412 OTU35
Tridentiger Tridentiger barbatus GOU103052 OTU187
Acentrogobius Acentrogobius caninus KY371079 OTU73
Acentrogobius Acentrogobius caninus GOU100542 OTU73
Parachaeturichthys Parachaeturichthys polynema BBG002013 OTU280
Cryptocentrus Cryptocentrus pavoninoides GOU102536 OTU144
Cryptocentrus Cryptocentrus pavoninoides GOU102537 OTU144
Cryptocentrus Cryptocentrus pavoninoides GOU102538 OTU144
Myersina Myersina crocata GOU102539 OTU145
Myersina Myersina filifer GOU102533 OTU143
Myersina Myersina filifer GOU102535 OTU143
Myersina Myersina filifer GOU102534 OTU143
Myersina Myersina filifer KY371771 OTU143
Myersina Myersina filifer KY371775 OTU143
Myersina Myersina filifer GOU103065 OTU143
Myersina Myersina filifer KY371776 OTU143
Myersina Myersina filifer KY371774 OTU143
Opistognathus Opistognathus macrolepis GOU101790 OTU132
Opistognathus Opistognathus macrolepis GOU101792 OTU132
Opistognathus Opistognathus macrolepis GOU101791 OTU132
Inegocia Platycephalus.sp GOU103016 OTU182
Acropoma Acropoma japonicum BBG020002 OTU283
Acropoma Acropoma japonicum BBG010001 OTU283
Acropoma Acropoma japonicum BBG025010 OTU283
Xiphasia Xiphasia setifer KY372382 OTU273
Rexea Rexea prometheoides KY372091 OTU251
Harpadon Harpadon nehereus GOU102961 OTU135
Harpadon Harpadon nehereus ZBL000621 OTU135
Harpadon Harpadon nehereus GOU101830 OTU135
Saurida Saurida elongata GOU100498 OTU49
Saurida Saurida tumbil BBG416001 OTU32
Saurida Saurida tumbil ZBL000943 OTU32
Saurida Saurida tumbil GOU100417 OTU32
Saurida Saurida tumbil BBG416009 OTU32
Saurida Saurida tumbil BBG416010 OTU32
Saurida Saurida tumbil ZBL000942 OTU32
Saurida Saurida tumbil ZBL000944 OTU32

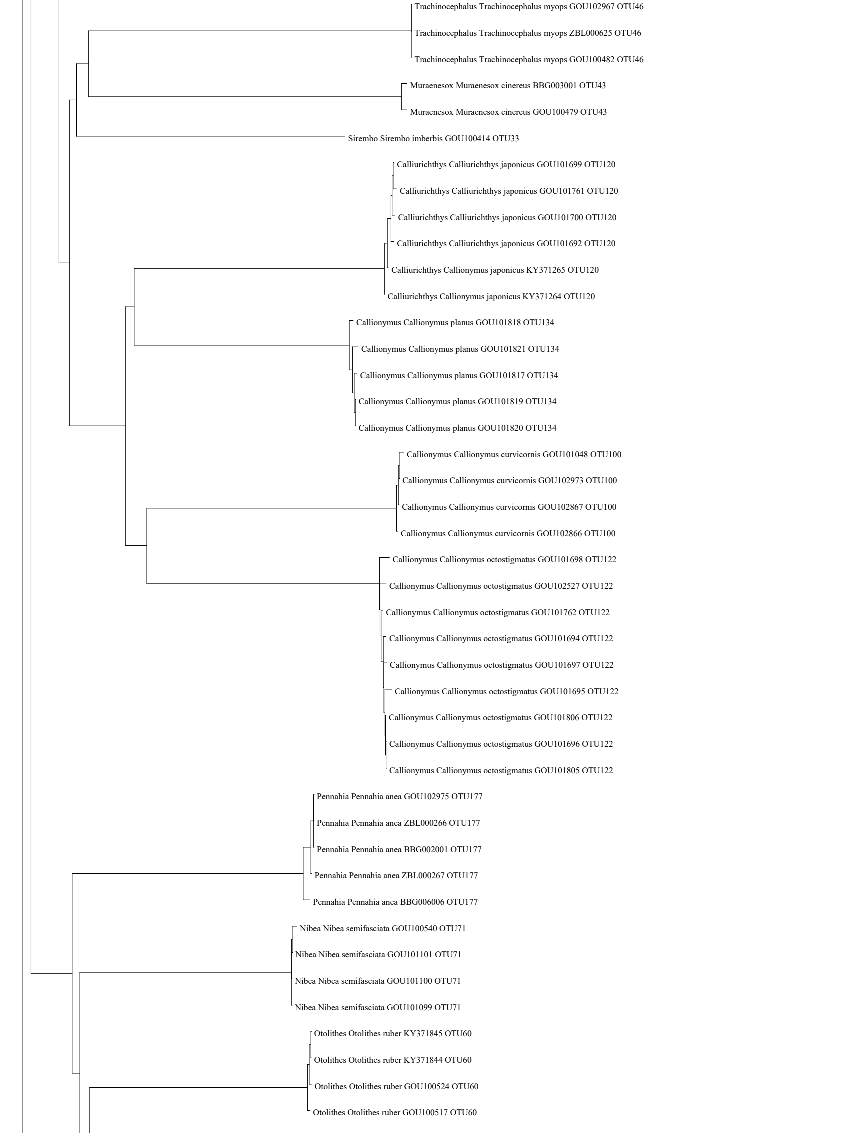
Trachinocephalus Trachinocephalus myops GOU102967 OTU46
Trachinocephalus Trachinocephalus myops ZBL000625 OTU46
Trachinocephalus Trachinocephalus myops GOU100482 OTU46
Muraenesox Muraenesox cinereus BBG003001 OTU43
Muraenesox Muraenesox cinereus GOU100479 OTU43
Sirembo Sirembo imberbis GOU100414 OTU33
Calliurichthys Calliurichthys japonicus GOU101699 OTU120
Calliurichthys Calliurichthys japonicus GOU101761 OTU120
Calliurichthys Calliurichthys japonicus GOU101700 OTU120
Calliurichthys Calliurichthys japonicus GOU101692 OTU120
Calliurichthys Callionymus japonicus KY371265 OTU120
Calliurichthys Callionymus japonicus KY371264 OTU120
Callionymus Callionymus planus GOU101818 OTU134
Callionymus Callionymus planus GOU101821 OTU134
Callionymus Callionymus planus GOU101817 OTU134
Callionymus Callionymus planus GOU101819 OTU134
Callionymus Callionymus planus GOU101820 OTU134
Callionymus Callionymus curvicornis GOU101048 OTU100
Callionymus Callionymus curvicornis GOU102973 OTU100
Callionymus Callionymus curvicornis GOU102867 OTU100
Callionymus Callionymus curvicornis GOU102866 OTU100
Callionymus Callionymus octostigmatus GOU101698 OTU122
Callionymus Callionymus octostigmatus GOU102527 OTU122
Callionymus Callionymus octostigmatus GOU101762 OTU122
Callionymus Callionymus octostigmatus GOU101694 OTU122
Callionymus Callionymus octostigmatus GOU101697 OTU122
Callionymus Callionymus octostigmatus GOU101695 OTU122
Callionymus Callionymus octostigmatus GOU101806 OTU122
Callionymus Callionymus octostigmatus GOU101696 OTU122
Callionymus Callionymus octostigmatus GOU101805 OTU122
Pennahia Pennahia anea GOU102975 OTU177
Pennahia Pennahia anea ZBL000266 OTU177
Pennahia Pennahia anea BBG002001 OTU177
Pennahia Pennahia anea ZBL000267 OTU177
Pennahia Pennahia anea BBG006006 OTU177
Nibea Nibea semifasciata GOU100540 OTU71
Nibea Nibea semifasciata GOU101101 OTU71
Nibea Nibea semifasciata GOU101100 OTU71
Nibea Nibea semifasciata GOU101099 OTU71
Otolithes Otolithes ruber KY371845 OTU60
Otolithes Otolithes ruber KY371844 OTU60
Otolithes Otolithes ruber GOU100524 OTU60
Otolithes Otolithes ruber GOU100517 OTU60

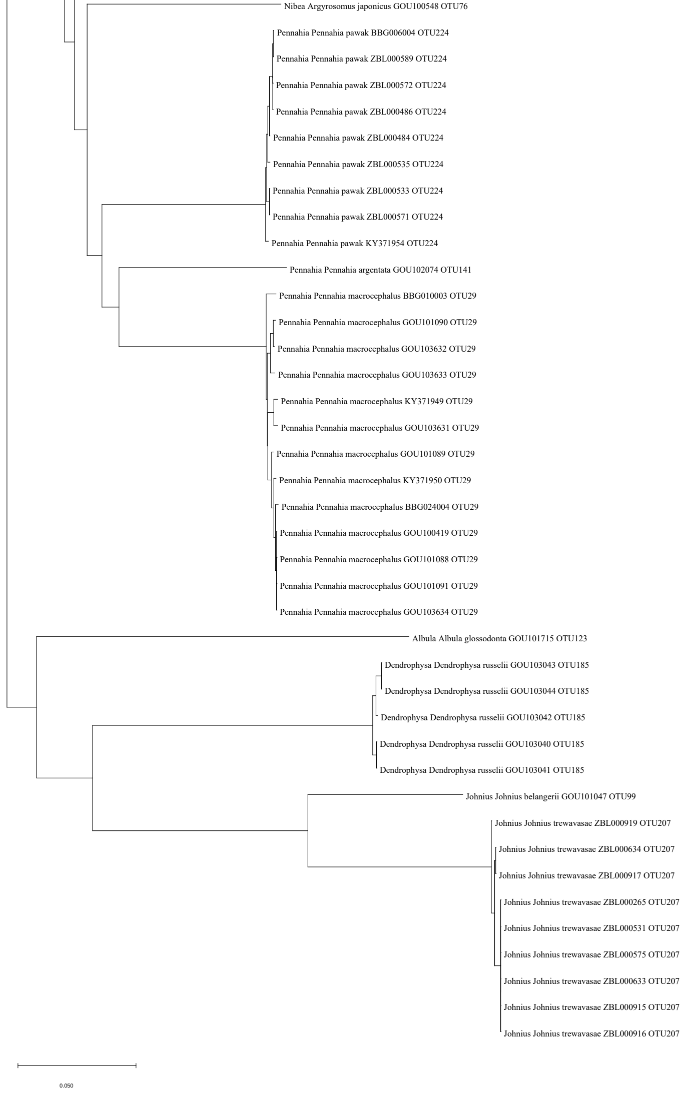

Nibea Argyrosomus japonicus GOU100548 OTU76
Pennahia Pennahia pawak BBG006004 OTU224
Pennahia Pennahia pawak ZBL000589 OTU224
Pennahia Pennahia pawak ZBL000572 OTU224
Pennahia Pennahia pawak ZBL000486 OTU224
Pennahia Pennahia pawak ZBL000484 OTU224
Pennahia Pennahia pawak ZBL000535 OTU224
Pennahia Pennahia pawak ZBL000533 OTU224
Pennahia Pennahia pawak ZBL000571 OTU224
Pennahia Pennahia pawak KY371954 OTU224
Pennahia Pennahia argentata GOU102074 OTU141
Pennahia Pennahia macrocephalus BBG010003 OTU29
Pennahia Pennahia macrocephalus GOU101090 OTU29
Pennahia Pennahia macrocephalus GOU103632 OTU29
Pennahia Pennahia macrocephalus GOU103633 OTU29
Pennahia Pennahia macrocephalus KY371949 OTU29
Pennahia Pennahia macrocephalus GOU103631 OTU29
Pennahia Pennahia macrocephalus GOU101089 OTU29
Pennahia Pennahia macrocephalus KY371950 OTU29
Pennahia Pennahia macrocephalus BBG024004 OTU29
Pennahia Pennahia macrocephalus GOU100419 OTU29
Pennahia Pennahia macrocephalus GOU101088 OTU29
Pennahia Pennahia macrocephalus GOU101091 OTU29
Pennahia Pennahia macrocephalus GOU103634 OTU29
Albula Albula glossodonta GOU101715 OTU123
Dendrophysa Dendrophysa russelii GOU103043 OTU185
Dendrophysa Dendrophysa russelii GOU103044 OTU185
Dendrophysa Dendrophysa russelii GOU103042 OTU185
Dendrophysa Dendrophysa russelii GOU103040 OTU185
Dendrophysa Dendrophysa russelii GOU103041 OTU185
Johnius Johnius belangerii GOU101047 OTU99
Johnius Johnius trewavasae ZBL000919 OTU207
Johnius Johnius trewavasae ZBL000634 OTU207
Johnius Johnius trewavasae ZBL000917 OTU207
Johnius Johnius trewavasae ZBL000265 OTU207
Johnius Johnius trewavasae ZBL000531 OTU207
Johnius Johnius trewavasae ZBL000575 OTU207
Johnius Johnius trewavasae ZBL000633 OTU207
Johnius Johnius trewavasae ZBL000915 OTU207
Johnius Johnius trewavasae ZBL000916 OTU207
0.050
